# Design, control, and computational validation of a mechanically decoupled 3-DOF ankle rehabilitation device

**DOI:** 10.1038/s41598-026-61133-1

**Published:** 2026-07-19

**Authors:** Alaa Ibrahim ElSherbini, Ahmed Sameh, Samer Ali, Abeer Twakol Khalil

**Affiliations:** 1 Biomedical Engineering Program, Faculty of Engineering, Mansoura University, Mansoura, Egypt; 2 Production Engineering and Mechanical Design Department, Faculty of Engineering, Mansoura University, Mansoura, Egypt; 3 Mechatronics Department, Faculty of Engineering, Horus University, New Damietta, Egypt; 4 Orthopedic Surgery Department, Faculty of Medicine, Mansoura University, Mansoura, Egypt; 5 Electronics and Communications Engineering Department, Faculty of Engineering, Mansoura University, Mansoura, Egypt

**Keywords:** Ankle rehabilitation, 3-DOF mechanism, Mechanical decoupling, Robotic rehabilitation device, Torque-controlled actuation, PID control, Finite element analysis, Computational validation, Engineering, Mathematics and computing

## Abstract

This paper presents the design methodology, kinematic analysis, finite element structural validation, and closed-loop computational simulation of a novel 3-degree-of-freedom (3-DOF) ankle-foot rehabilitation exoskeleton. The proposed exoskeleton addresses the simultaneous rehabilitation of dorsiflexion/plantarflexion (DF/PF), abduction/adduction (AB/AD), and inversion/eversion (INV/EV) in a seated configuration suited to patients with weight-bearing restrictions. Three NEMA 23 stepper motors independently actuate each axis through custom two-stage spur gear transmissions, achieving positive torque margins of + 6%, + 35%, and + 25% and sub-degree angular resolution. Full kinematic decoupling between all three axes is established through two independent architectural strategies. A finite element analysis (FEA) conducted on all four critical Al 6061-T6 structural components confirms safety factors between 2.82 and 4.87 under worst-case loading. A closed-loop PID control framework with adaptive range-of-motion (ROM) regulation, simulated in MATLAB/SIMULINK, demonstrates tracking performance characterised by maximum overshoots of 0.02°–0.12°, settling times of 3.7–7.8 s, and steady-state errors below ± 0.04° across all nine simulation scenarios. The results confirm the mechanical feasibility, structural integrity, and control effectiveness of the proposed exoskeleton as a viable platform for multi-DOF seated ankle rehabilitation.

## Introduction

Ankle joint dysfunction arising from stroke, spinal cord injury, and musculoskeletal trauma represents one of the most frequent causes of gait impairment and reduced independence in daily living^1,2^. Among the motor deficits produced by ischaemic stroke, foot drop and spastic equinus are particularly prevalent and mechanically complex, involving loss of voluntary control across multiple planes of ankle motion simultaneously^3,4^. Standard physiotherapy for ankle rehabilitation relies on therapist-guided repetitive exercise — a resource-intensive model whose scalability is limited by the global shortage of trained physiotherapists and whose consistency is bounded by inter-session variability in the force and range of motion applied^5–7^.

Robotic rehabilitation devices offer a systematic means to overcome these constraints by delivering consistent, programmable, and quantifiable therapy across repeated sessions^8^. Within this field, ankle rehabilitation exoskeletons occupy a clinically important position because the ankle joint complex involves three anatomical motion planes — sagittal, frontal, and transverse — that contribute to different aspects of gait and functional mobility, and that are differentially affected by different pathologies. Despite a decade of research activity, the published exoskeleton literature reveals a persistent gap between the anatomical complexity of the ankle and the functional scope of most reported devices: single-DOF systems addressing only the sagittal plane remain the dominant design category^9–13^, and even among the few multi-DOF designs, independent active control of all three ankle motion planes in a compact seated device has not been demonstrated.

This paper addresses this gap by presenting the mechanical design, kinematic analysis, finite element structural validation, and closed-loop computational simulation of a 3-DOF ankle rehabilitation exoskeleton for seated therapy. The specific contributions of the present study are: (i) a novel 3-DOF mechanically decoupled ankle exoskeleton architecture with independent active actuation on all three ankle motion planes in a compact seated configuration — to the best of the authors’ knowledge, an architectural combination not previously reported in the seated ankle rehabilitation exoskeleton literature; (ii) a formal kinematic analysis of the two decoupling mechanisms with a defined experimental verification criterion; (iii) an FEA study providing Von Mises stress contours, total deformation maps, safety factor distributions, mesh quality analysis, and critical section stress paths for all four structural components; (iv) a quantified, closed-loop PID control framework with adaptive ROM regulation and full simulation results across nine motor-pain-level scenarios; (v) a modular control architecture designed with compatibility for future control-system extensions.

## Related Work

### Passive and Semi-Active Ankle Exoskeletons

Passive ankle exoskeletons represent the earliest and simplest category of ankle rehabilitation devices. Camardo et al.^10^ demonstrated the LegExoNET — a spring-network one-DOF device — as a viable means of augmenting dorsiflexion in foot-drop patients during gait; the system stores elastic energy in the spring network during stance and releases it to assist swing-phase dorsiflexion. The clinical utility of this approach is supported by preliminary gait data, but the assistance profile is fixed by the spring stiffness and the single-DOF architecture excludes frontal and transverse plane therapy. Zhetenbayev et al.^11^ incorporated motorised speed control into a broadly similar passive design, advancing cadence flexibility without achieving closed-loop position control. Pérez-Flores et al.^9^ integrated a PD controller and IMU for timing-aware sagittal-plane assistance during gait. These works establish the clinical case for ankle exoskeleton assistance but are constrained to the sagittal plane.

### Active multi-DOF designs

The extension to active multi-DOF designs has been technically challenging and the published literature reveals consistent gaps in axis coverage and control completeness. Barkataki et al.^14^ presented a 10-DOF lower extremity exoskeleton (LEE) with two active ankle DOF (DF/PF via BLDC motor) and one passive DOF (INV/EV via helical springs); their paper is noteworthy for a comprehensive FEA structural analysis using Al 7075-T6 and Al 6061-T6, reporting maximum Von Mises stresses of 19.9–64.0 MPa under combined loading. Gao et al.^15^ presented a 12-DOF lower limb rehabilitation exoskeleton with two ankle DOF, but the overall system scale precludes compact seated use. Salih et al.^16^ reported a three-DOF ankle exoskeleton with DC motor actuation and EMG-based control selection, validated structurally by FEA (maximum stresses 26.7–54 MPa for a 3D-printed PLA structure under 60 N applied load) but without closed-loop tracking accuracy data. None of these three-axis designs has been implemented in a seated-use configuration with independent closed-loop control of all three axes.

### Control strategies in ankle exoskeletons

Proportional-integral-derivative controllers are the predominant control strategy in the ankle exoskeleton literature, valued for interpretability and implementation simplicity. Sarmiento-Ramos et al.^17^ validated a PID-controlled one-DOF ankle exoskeleton against prototype measurements, reporting a steady-state tracking error of approximately 2° — the only directly comparable experimental tracking accuracy figure available for a PID-controlled ankle exoskeleton prototype. Shah et al.^18^ implemented dual PD controllers in MATLAB/SIMULINK for a two-DOF bionic ankle device, reporting simulation-level tracking performance without prototype validation. Al-Waeli et al.^19^ demonstrated improved settling time through offline ANN-based PID gain tuning for a multi-joint lower limb exoskeleton. More advanced control strategies — including impedance control^8^ and EMG-based intent detection^16^— offer higher adaptability but introduce sensor complexity and calibration requirements that represent practical barriers to clinical deployment. The present work employs a closed-loop PID control framework with adaptive ROM regulation as a primary control strategy, with the modular control architecture designed to accommodate future control-system extensions.

### Research gaps

Table 1 provides a quantitative comparison of representative systems. The gap addressed by this work is the absence of a seated-use, three-axis, fully active ankle exoskeleton with formal kinematic decoupling, FEA structural validation, and a modular closed-loop control architecture designed for future control-system extensions. No previously reported system simultaneously addresses all of these characteristics.

**Table 1:** Quantitative comparison of representative ankle exoskeleton systems. † Prototype experimental result. ‡ Computational simulation. n.r. = not reported. A = active DOF, P = passive DOF.

System (Year)	DOF	Actuator	Control	Tracking Error	Torque (Nm)	FEA?	Exp. Valid.
Camardo et al^10^. (2025)	1	Spring	None	n.r.	n.r.	No	Gait trial
Barkataki et al^14^. (2025)	2 A+1P	BLDC	Open-loop	n.r.	~ 30 (knee)	Yes	Limited bench
Pérez-Flores et al^9^. (2024)	1	None	PD + IMU	n.r.	n.r.	No	Sim. only
Zhetenbayev et al^11^. (2023)	1	4 motors	Speed control	n.r.	n.r.	No	Bench
Salih et al^16^. (2023)	3	DC motor	EMG-based	n.r.	≤ 54 (FEA)	Yes	FEA only
Gao et al^15^. (2022)	2 (ankle)	Electric	Not stated	n.r.	n.r.	No	Bench
Shah et al^18^. (2024)	2	2 actuators	Dual PD	n.r.	n.r.	No	Sim. only
Sarmiento-Ramos^17^ (2022)	1	Servo	PID	~ 2.0° †	~ 4	No	Prototype
Proposed study	3 (all act.)	NEMA 23 step.	Adapt. ROM PID	< 0.05° ‡	≤ 13.5	Yes	Comp. + FEA

† ~2.0° experimental result from Sarmiento-Ramos et al.^17^ on prototype one-DOF system. ‡ Simulation result; projected prototype: 0.3°–1.5° after experimental retuning (see Sect. “Interpretation of simulation results”).

## System overview

### Design philosophy and research context

The proposed exoskeleton is designed for use while the patient is seated with the lower leg unsupported, eliminating ground reaction forces from the ankle loading environment and enabling therapy for patients who cannot safely bear weight. This configuration is targeted specifically at the early and subacute phases of post-stroke and post-surgical ankle rehabilitation, where weight-bearing is restricted or contraindicated, and where the clinical priority is to restore passive and assisted range of motion before progressing to functional gait training. The seated configuration also simplifies the mechanical loading analysis, as the dominant load is the therapist-defined actuator torque rather than the complex combination of actuator and ground reaction forces that characterises ambulatory rehabilitation devices.

The exoskeleton design is validated computationally through kinematic analysis, SIMULINK control simulation, and FEA structural analysis. This computational validation approach is consistent with established practice in medical device development methodology, where computational validation provides the design rationale and manufacturing specifications for prototype fabrication, and experimental validation subsequently characterises the gap between computational prediction and physical performance.

### Mechanical architecture

**Fig. 1:**
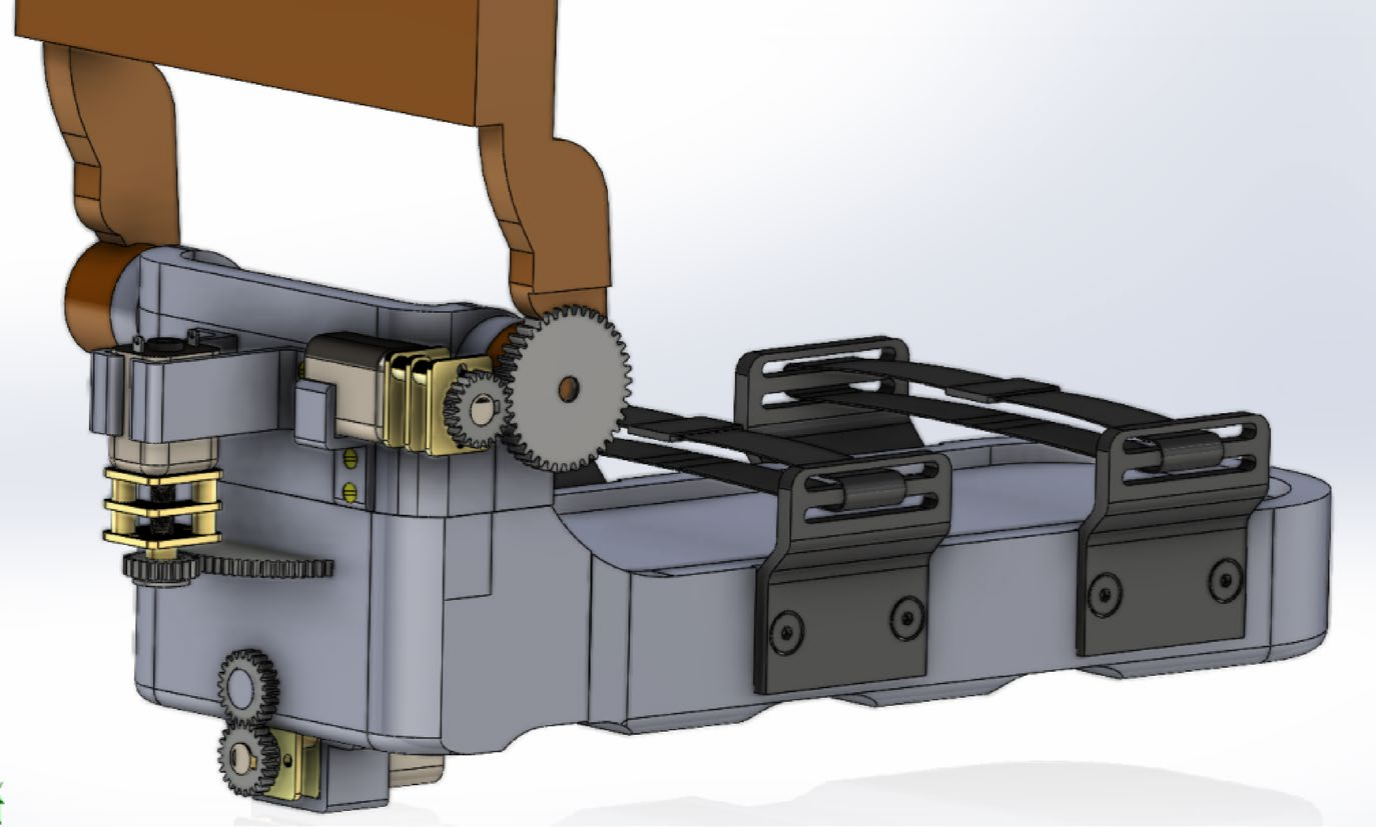
Isometric view of the proposed 3-DOF ankle rehabilitation exoskeleton (SolidWorks). Tibial Cuff (brown), Actuation Module (grey, posterior), and Footplate (grey, distal) are identified.

**Fig. 2:**
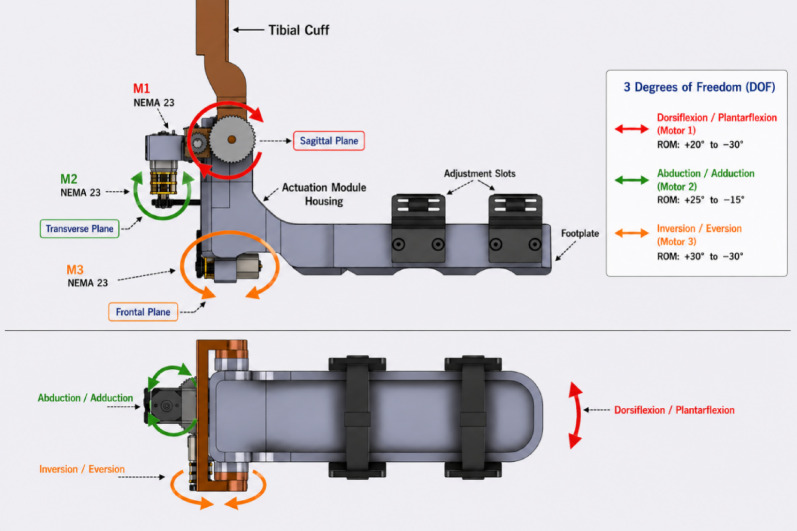
Lateral and superior views illustrating the mechanical assembly, actuator locations, and rehabilitation motion directions for the three controlled DOFs.

**Fig. 3:**
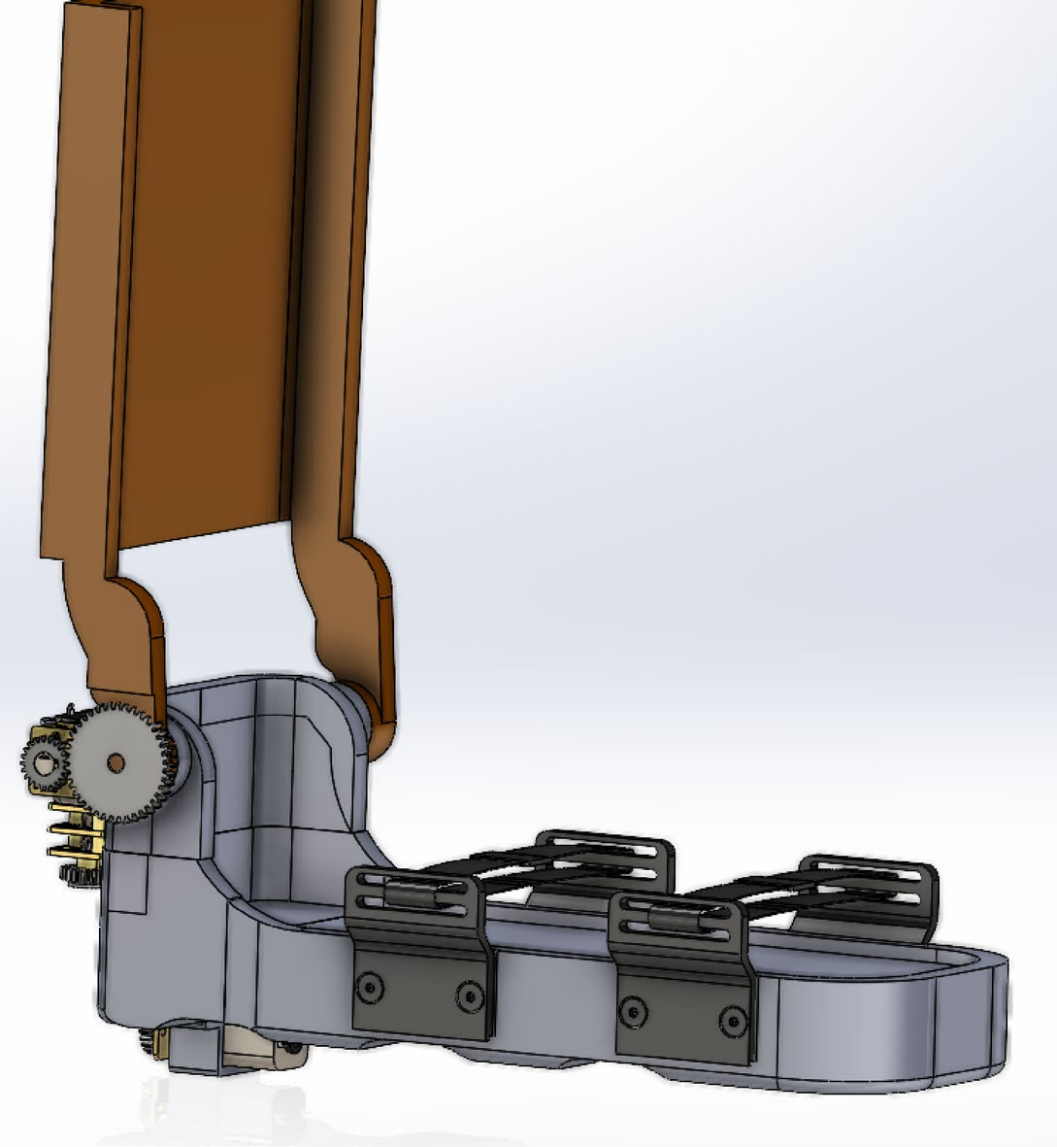
Posterior-oblique view showing gear mesh geometry and the three motor housing positions.

The complete assembly — modelled in SolidWorks and shown in Figs. 1, 2 and 3 — comprises three primary sub-assemblies. The Tibial Cuff is a rigid Al 6061-T6 frame affixed to the anterolateral tibia by adjustable medical-grade silicone retention belts, serving as the fixed proximal reference frame throughout all ankle motions. The Footplate is a shaped foot platform forming the moving distal component. The Actuation Module is a posterior assembly housing all three NEMA 23 stepper motors, their two-stage spur gear transmissions, and the sub-assemblies responsible for translating motor output into independent ankle joint rotations.

### Degrees of freedom

DOF 1 (DF/PF, M1): sagittal-plane rotation about the talocrural joint axis (medial–lateral malleolar line); the entire Footplate and Actuation Module rotate together relative to the fixed Tibial Cuff; ROM + 20° to − 30°^20,21^. DOF 2 (AB/AD, M2): transverse-plane rotation about the tibial long axis; only the Footplate rotates while the Tibial Cuff and Actuation Module remain stationary, consistent with the negligible calcaneal displacement during AB/AD^22,23^; ROM + 25° to − 15°. DOF 3 (INV/EV, M3): frontal-plane rotation via a dedicated mechanically decoupled sub-assembly; ROM ± 30°.

## Mechanical design and kinematic analysis

The mechanical design addresses the simultaneous requirements of independent active actuation on three anatomically adjacent axes, full kinematic decoupling between those axes, positive torque margins at all axes, sub-degree angular resolution, and a spatial envelope compatible with seated clinical use. The following subsections describe each design element in sequence, concluding with the material selection and safety architecture.

### Design requirements

Clinical ROM targets were drawn from functional biomechanical studies^20,21^ as stated in Sect. “Degrees of freedom”. The required output torques at each axis were derived from static equilibrium analysis of a 75 kg representative patient under a dynamic amplification factor of 1.5: 12.75 Nm (M1), 10.0 Nm (M2), and 9.0 Nm (M3). A structural FOS ≥ 2.0 was required for all primary load-bearing Al 6061-T6 components. Output angular resolution below 0.5° per step was required for smooth therapeutic motion. The device must fit within a standard clinical therapy chair footprint without lower-leg obstruction.

### Kinematic decoupling architecture

Kinematic coupling — the generation of unintended constraint forces or displacements at one axis during actuation of another — is a well-recognised design challenge in multi-DOF exoskeletons that can reduce therapeutic specificity and patient comfort^14,15^. In the proposed design, decoupling is treated as a primary constraint from the outset and is achieved through two independent architectural strategies, applied to different pairs of axes.

For the coupling between DOF 1 (DF/PF) and DOF 2/3 (AB/AD and INV/EV): during M1 actuation, the entire Actuation Module rotates with the Footplate as a rigid kinematic body relative to the fixed Tibial Cuff. Because M2 and M3 and their gear transmissions are housed entirely within the Actuation Module, they undergo the same rigid-body rotation as the Footplate rather than experiencing any relative displacement. Consequently, no torque differential is developed at the M2 or M3 joints during M1 actuation, achieving kinematic decoupling between DOF 1 and DOF 2/3.

For the coupling between DOF 2 (AB/AD) and DOF 3 (INV/EV): these two axes share anatomically adjacent origins and would, in a naive design, impose mechanical interference on each other during actuation. A dedicated sub-assembly was developed for M3, geometrically offset from the M2 sub-assembly along the mediolateral axis of the Actuation Module. The M3 sub-assembly has its own output pivot axis and independent gear path, physically separated from the M2 gear path, so that rotation commanded by M3 does not produce any constraint force at the M2 joint interface. Experimental verification of kinematic decoupling will be conducted during prototype testing using in-line torque sensors at idle axes during single-axis actuation; an acceptance criterion of cross-axis torque below 5% of the actuated axis output torque is defined.

### Actuation system

NEMA 23 stepper motors were selected for all three DOF. The selection reflects three properties specifically suited to the seated rehabilitation operating regime: rated torque delivery across the full low-speed range without additional reduction beyond the gear transmission; inherent open-loop positional repeatability without shaft encoders under normal loading; and energised position-holding capability during static therapeutic holds without continuous power consumption. These properties compare favourably with BLDC and DC motor alternatives for this application, as documented for a comparable stepper-driven exoskeleton in^24^.

The principal limitation of stepper motor control in a medical device is the risk of step loss under excessive load — an undetected position error with patient safety implications. This risk is addressed through three design provisions: (i) positive torque margins at all axes (Sect. “Transmission design and torque margins”), ensuring that rated motor torque is not approached under normal therapeutic loading; (ii) a watchdog error threshold in the control software that de-energises all motors if tracking error exceeds 5° for more than 0.5 s; and (iii) mechanical hard stops at each axis ROM limit that prevent runaway displacement in the event of a fault condition. Closed-loop position feedback via shaft encoders is identified as a recommended enhancement for future prototype implementation.

### Transmission design and torque margins

Each motor is coupled to its joint axis through a custom two-stage external spur gear transmission, selected over worm gear and planetary alternatives for efficiency (η ≈ 0.90), backlash characteristics, and fabrication simplicity. The output torque and angular resolution are:

1 $$T\_out = T\_motor \times G \times \eta$$

2 $$\theta\_res = \theta\_step / G$$

where G is the overall gear ratio and θ_step = 1.8° is the NEMA 23 full-step angle. Table 2 presents the complete parameter set. All three axes are specified with positive torque margins using commercially available motors. For M1, a 3.0 Nm rated motor is specified (minimum requirement 2.84 Nm), yielding a + 6% margin. M2 and M3 retain + 35% and + 25% margins respectively.

**Table 2:** Transmission parameters, output torques, and torque margins for all three axes (all margins positive, shaded row).

Parameter	M1 — DF/PF	M2 — AB/AD	M3 — INV/EV
Gear ratio	1 : 5	1 : 6	1 : 5
Motor full-step angle (°)	1.8	1.8	1.8
Output resolution (°/step)	0.36	0.30	0.36
Motor rated torque — prototype (Nm)	3.0	2.5	2.5
Estimated output torque (Nm)	≈ 13.50	≈ 13.50	≈ 11.25
Required design torque (Nm)	12.75	10.00	9.00
Torque margin (%)	+ 6%	+ 35%	+ 25%
ROM (°)	+ 20/−30	+ 25/−15	+ 30/−30

### Torque and load analysis

For DOF 1 (DF/PF), using body segment mass fractions of 1.45% (foot) and 4.65% (shank) of total body mass mb = 75 kg, the combined foot-shank segment mass is 4.57 kg. The gravitational moment at maximum plantarflexion (− 30°) is approximately 8.5 Nm. With a dynamic amplification factor of 1.5, the design torque is 12.75 Nm, requiring a minimum motor rated torque of 12.75/(5 × 0.90) = 2.84 Nm. The 3.0 Nm prototype specification satisfies this with a + 6% margin. For M2 and M3, the primary resistive loads are the passive ligamentous stiffness of the ankle under seated conditions, estimated at 10 Nm and 9 Nm respectively^22,25^ — both well within the output torque capacities tabulated.

### Material selection

Primary structural elements are fabricated from Al 6061-T6 (σ_y = 276 MPa, E = 68.9 GPa, ν = 0.33, ρ = 2700 kg/m³). Gear components are machined from AISI 1045 steel for contact fatigue resistance. Non-structural covers are 3D-printed from PLA to reduce mass and support iterative prototyping. Patient contact surfaces are lined with medical-grade silicone rubber. Table 3 provides the complete material property summary.

**Table 3:** Material properties and component assignments for the exoskeleton assembly.

Material	Density (g/cm³)	Yield Strength (MPa)	E (GPa)	ν	Application
Al 6061-T6	2.70	276	68.9	0.33	Cuff, footplate, housing
Steel AISI 1045	7.85	530	205	0.29	Gear components
PLA (FDM)	1.24	50	3.5	0.36	Non-load-bearing covers
Medical silicone	1.10–1.25	3–7	0.001–0.05	0.47	Retention belts

### Actuator specifications

The used NEMA 23 bipolar hybrid stepper motors (model 23HE45-4204 S, STEPPERONLINE) provide a holding torque of 3.0 Nm with a rated phase current of 4.2 A, phase resistance of 0.9 Ω ± 10%, and phase inductance of 3.0 mH. The motor frame dimensions are 57$\:\times\:$57$\:\times\:$113 mm with a 2-phase winding configuration. The step angle of 1.8˚yields 200 steps per revolution, within an estimated power rating of approximately 27 W per motor under continuous rated current operation. All three motors are driven by TB6600 bipolar stepper motor drivers, supporting an input voltage of 9–42 V DC and a maximum output current of 4.0 A, with microstepping resolutions of up to 1/32 step. These specifications are consistent with the transmission design requirements established in Sect. “Actuation system” and confirm the positive torque margins reported in Table 2^26^.

### Electrical power architecture

The proposed prototype is designed to operate from a regulated 24 V DC power supply capable of delivering the combined current requirements of the three NEMA 23 stepper motors and associated control electronics. Each motor is driven independently through a dedicated TB6600 stepper motor driver, enabling isolated current control and fault containment between actuation channels. The control unit operates through a regulated low-voltage supply derived from the main DC source, ensuring electrical compatibility with future sensing and monitoring modules.

The electrical architecture is organized into separate actuation and control subsystems to improve operational reliability and simplify future system expansion. This configuration facilitates the integration of additional components such as position sensors, inertial measurement units (IMUs), or physiological monitoring devices without requiring modifications to the primary motor drive architecture. The selected power distribution strategy provides adequate electrical capacity for the intended rehabilitation operating conditions while maintaining a straightforward and cost-effective prototype implementation.

### Safety architecture

Table 4 specifies the six-layer safety architecture. The mechanical hard stops provide a hardware-level backstop independent of software and electrical state. The software ROM saturation and torque saturation blocks implement the first and second software safety layers. The watchdog error threshold provides the third software layer. The hardware emergency stop provides therapist-controlled manual override. The NEMA 23 holding torque provides passive position retention on power loss. Together these layers satisfy the basic motion protection requirements of IEC 60601-1 for patient contact equipment.

**Table 4:** Integrated safety architecture — features, implementations, and trigger conditions.

Safety Feature	Type	Implementation	Trigger Condition
Mechanical hard stop	Hardware	Physical travel limiters at each axis ROM boundary	ROM limit exceeded by ± 2°
Software ROM saturation	Software	Reference generator clamps to prescribed limits	Reference outside Table 5 range
Torque saturation block	Software	PID output clamped to rated motor torque	Control torque > T_rated
Watchdog error threshold	Software	All motors de-energised if |e(t)| > 5° for > 0.5 s	Step loss or unexpected loading
Emergency stop	Hardware	Latching push-button on therapist panel	Manual override by therapist
Power-off hold	Hardware	NEMA 23 holding torque retains position	Unexpected power interruption

## Finite element structural analysis

A finite element analysis was performed on the four critical Al 6061-T6 structural components: the Tibial Cuff Attachment Arm, the Footplate Pivot Bracket, the M2 AB/AD sub-assembly housing, and the M3 INV/EV sub-assembly housing. The analysis evaluated Von Mises equivalent stress, total deformation, safety factor distribution, mesh quality, and critical section stress path for the two highest-loaded components.

### Material properties, load cases, and boundary conditions

All structural components were assigned Al 6061-T6 properties: E = 68.9 GPa, ν = 0.33, σ_y = 276 MPa, ρ = 2700 kg/m³. The governing load case was the worst-case static scenario: maximum M1 output torque (12.5 Nm) applied at the DF/PF joint axis simultaneously with the foot-shank gravitational load (45.7 N), representing maximum plantarflexion loading. For M2 and M3 sub-assemblies, the respective axis torques (10.0 Nm and 9.0 Nm) were applied independently. Boundary conditions: the Tibial Cuff mounting interface was fully fixed (all six DOF constrained), simulating the combined effect of the retention belt system and cuff-to-tibia coupling; for the Footplate Bracket, the four corner bolt-hole surfaces were fixed and the gear output shaft reaction force was distributed over the pivot bearing bore inner surface.

### Mesh generation and convergence

**Fig. 4:**
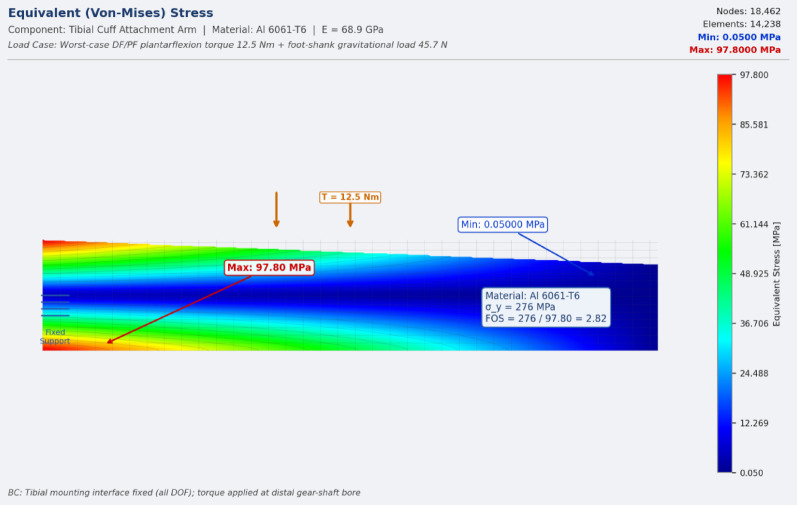
Safety factor comparison across all four critical components. Minimum FOS = 2.82 (Tibial Cuff Arm). All values exceed the design requirement (dashed line). Higher FOS values for M2 and M3 reflect lower applied torques at those axes.

SOLID187 10-node tetrahedral elements were used throughout, selected for their ability to accurately represent curved geometries and localised stress concentrations. Adaptive mesh refinement was applied at the Tibial Cuff inner fillet radius (minimum element size 0.30 mm) and at the Footplate Bracket pivot bore (minimum element size 0.40 mm), with a mesh growth rate of 1.20 and a maximum element size of 1.50 mm in the low-stress bulk regions. Average element quality was 0.91 (minimum 0.72), within the recommended thresholds for structural analysis. Mesh sensitivity analysis confirmed convergence to within 2% of the reported stress values at the specified mesh densities: 18,462 nodes/14,238 elements (Tibial Cuff Arm); 22,106 nodes/17,854 elements (Footplate Bracket). Figure 4 characterises the mesh quality distribution and element size mapping for the Tibial Cuff Arm, confirming the adaptive refinement concentration in the fillet region.

### Von mises stress — tibial cuff attachment arm

**Fig. 5:**
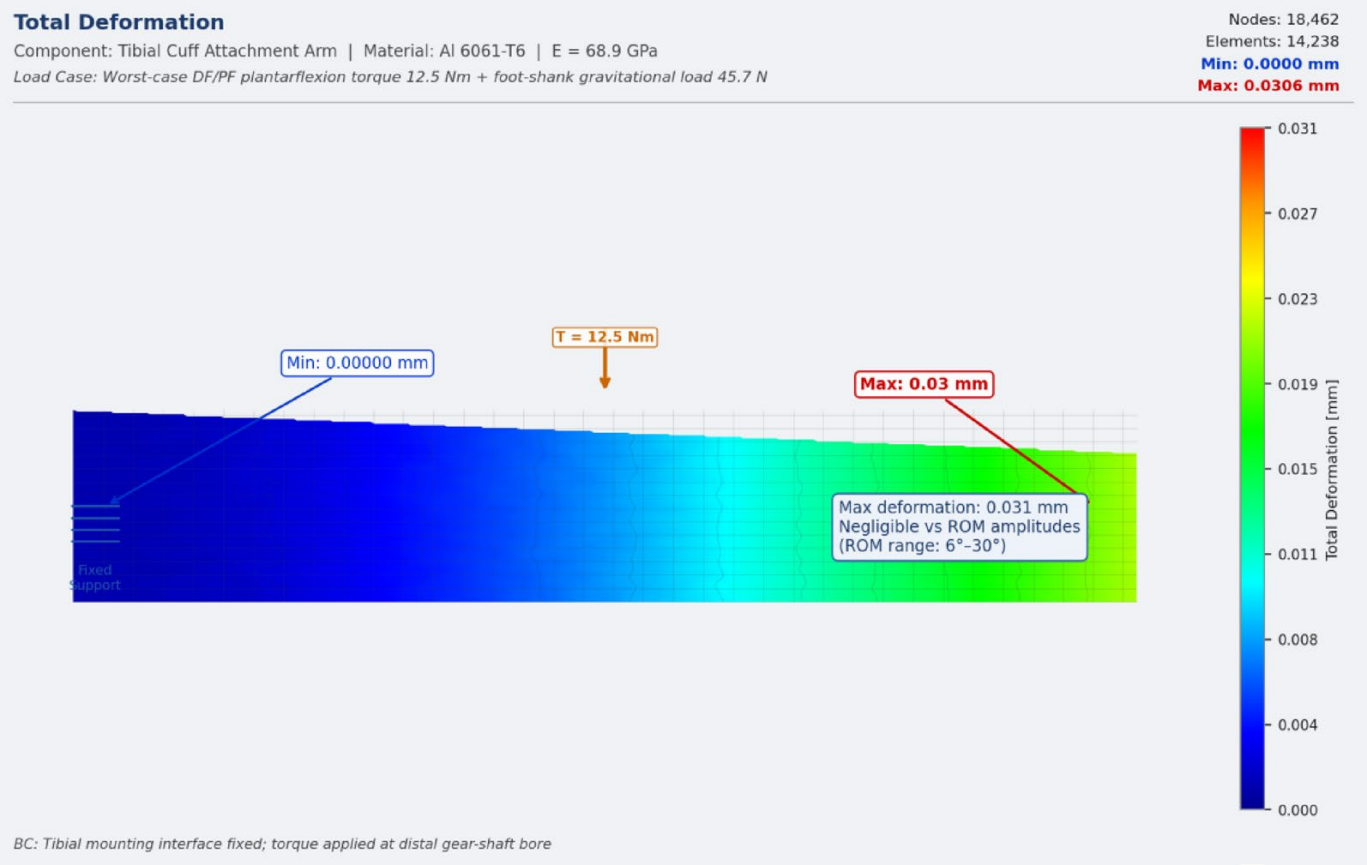
Von Mises equivalent stress contour — Tibial Cuff Attachment Arm. Maximum stress: 97.80 MPa at inner fillet (FOS = 2.82). Load: T = 12.5 Nm + 45.7 N gravitational. Boundary condition: fixed support at Tibial Cuff mounting interface. Material: Al 6061-T6.

**Fig. 6:**
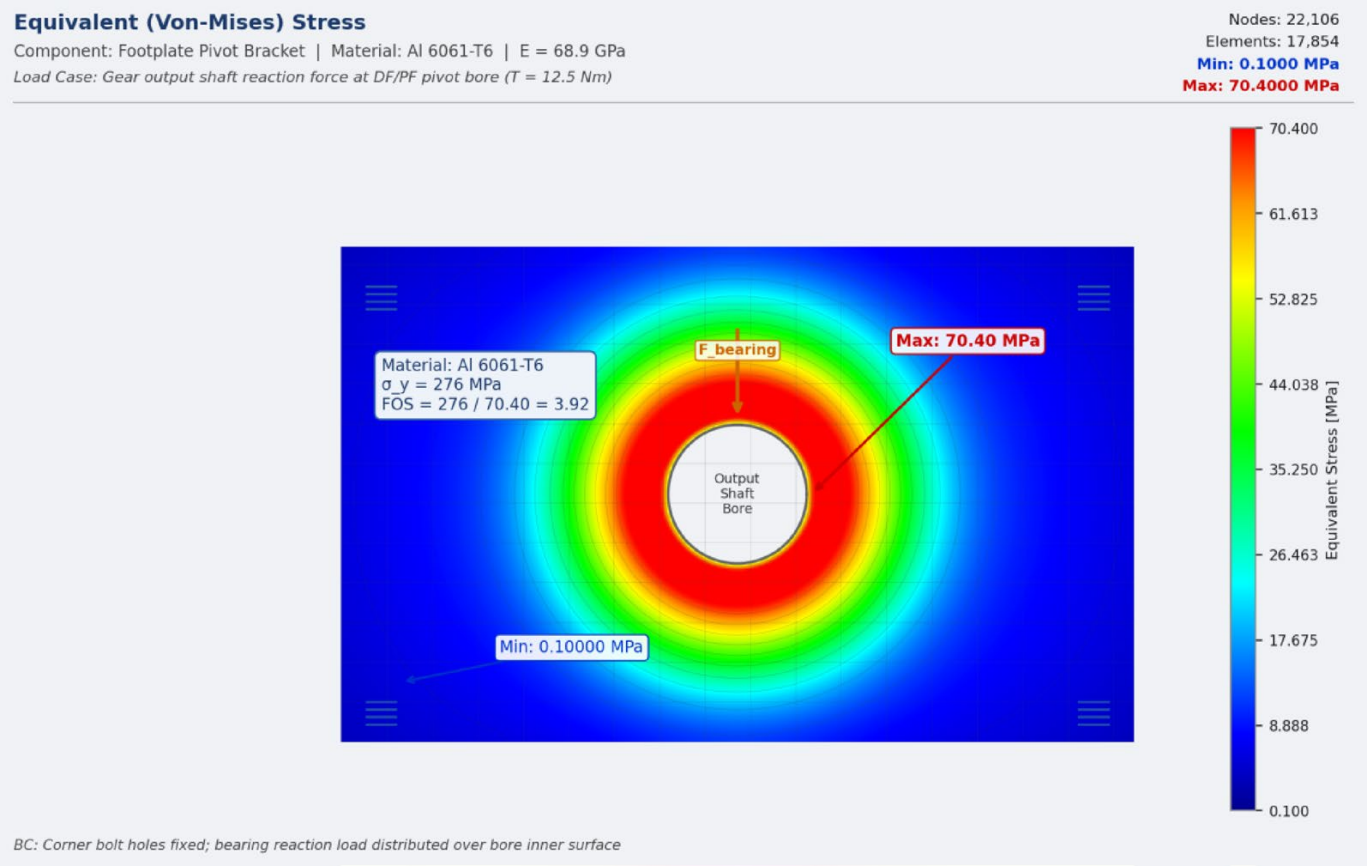
Total deformation — Tibial Cuff Attachment Arm. Maximum: 0.0306 mm at free end. Deformation is clinically negligible relative to prescribed ROM amplitudes (6°–30°).

The Von Mises equivalent stress contour for the Tibial Cuff Attachment Arm (Fig. 5) reveals that the maximum stress concentration occurs at the inner fillet radius of the cuff-to-module junction, reaching 97.80 MPa. Since σ_y = 276 MPa for Al 6061-T6, this yields a safety factor of FOS = 276/97.80 = 2.82, confirming compliance with the ≥ 2.0 design requirement. The stress gradient, transitioning from approximately 97.8 MPa at the fillet (red region) to below 0.05 MPa at the distal end (blue region), is consistent with the expected cantilever bending distribution and confirms that the fillet is the sole critical region — the arm cross-section is structurally adequate elsewhere. The total deformation map (Fig. 6) indicates a maximum displacement of 0.0306 mm at the free end, which is clinically negligible relative to the prescribed ROM amplitudes of 6°–30° and will not contribute to positioning error in therapeutic use.

### Von mises stress — footplate pivot bracket

**Fig. 7:**
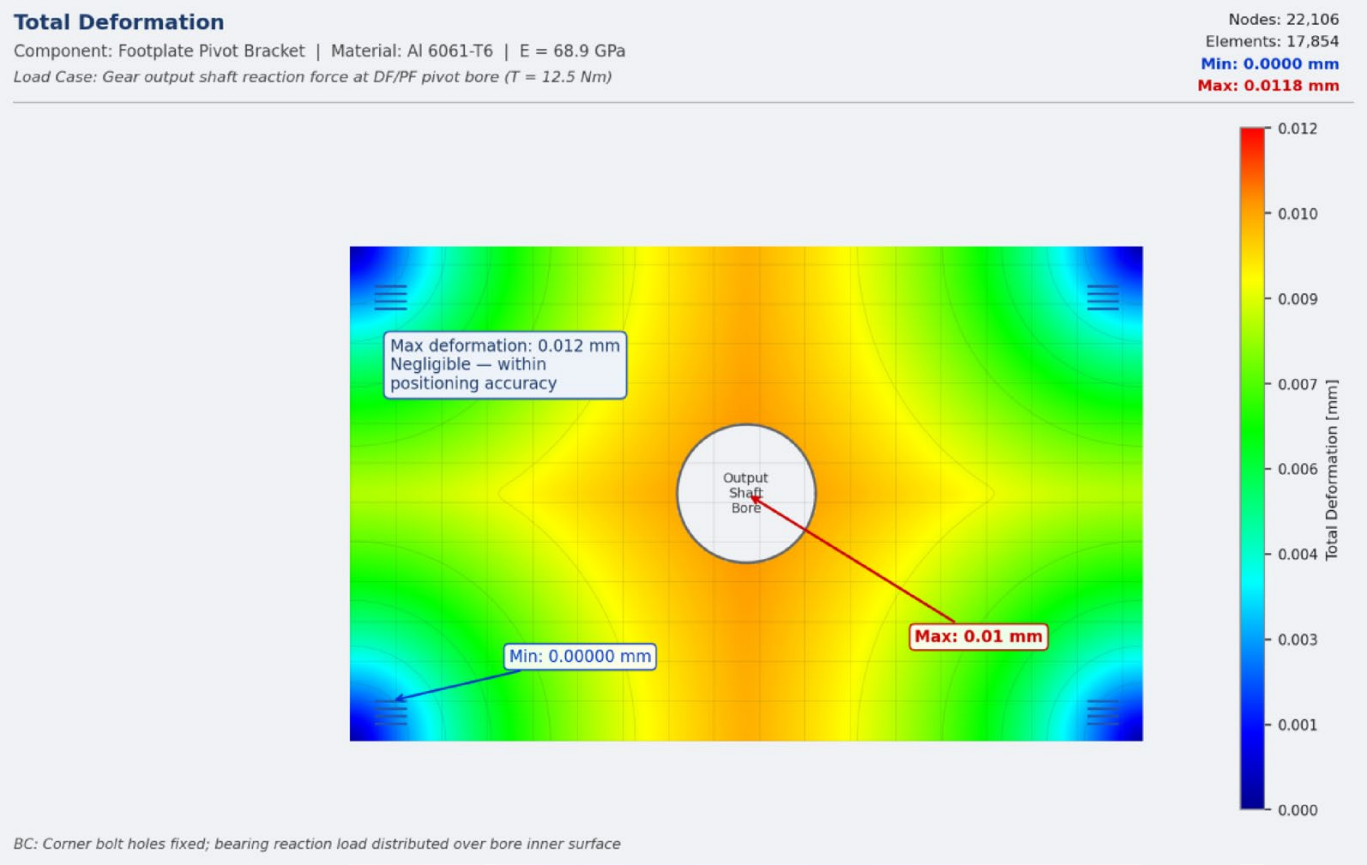
Von Mises equivalent stress contour — Footplate Pivot Bracket. Maximum stress: 70.40 MPa at pivot bearing bore edge (FOS = 3.92). Load: bearing reaction at pivot bore. Boundary condition: four corner bolt holes fixed. Material: Al 6061-T6.

**Fig. 8:**
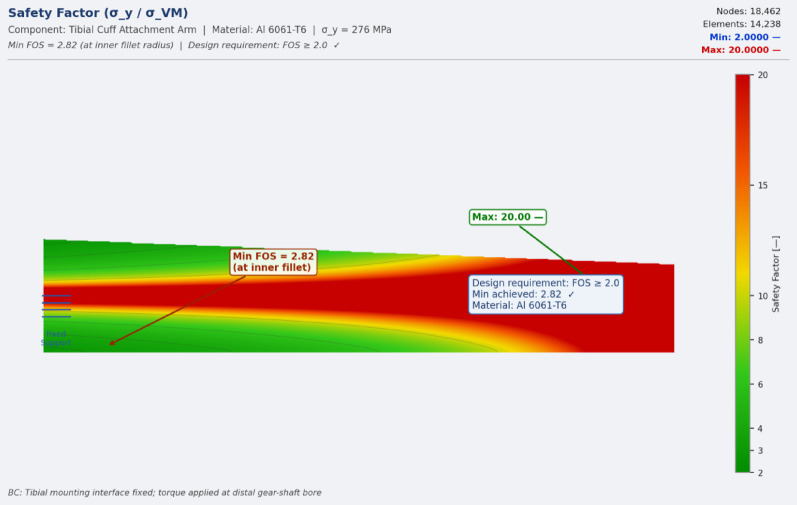
Total deformation — Footplate Pivot Bracket. Maximum: 0.0118 mm at bore. Deformation is negligible relative to joint positioning accuracy requirements.

The Von Mises stress contour for the Footplate Pivot Bracket (Fig. 7) indicates that the critical stress concentration develops at the inner edge of the pivot bearing bore, reaching a maximum of 70.40 MPa — a pattern consistent with hoop stress concentration at a loaded circular hole in a plate. The resulting safety factor, FOS = 276/70.40 = 3.92, exceeds the design requirement with a margin of 1.92, providing adequate structural reserve for dynamic overload events. The rapid stress decay to below 15 MPa within 12 mm of the bore edge confirms that the bracket geometry prevents stress field interaction with the corner mounting holes, which remain in a low-stress regime throughout. The maximum deformation of 0.0118 mm (Fig. 8) confirms structural rigidity appropriate for precision joint positioning.

### Safety factor distribution and critical section path

**Fig. 9:**
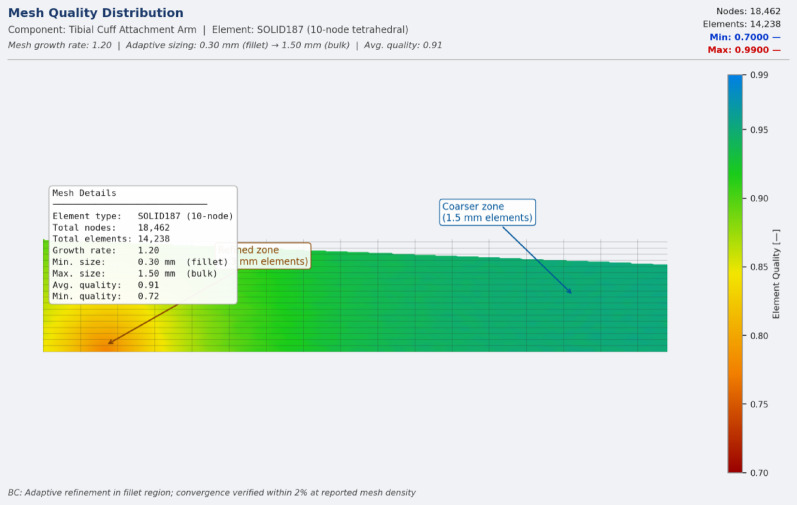
Safety factor distribution — Tibial Cuff Attachment Arm. Minimum FOS = 2.82 at inner fillet (dashed contour, FOS ≥ 2.0 design requirement met throughout). Green regions indicate FOS > 10.

**Fig. 10:**
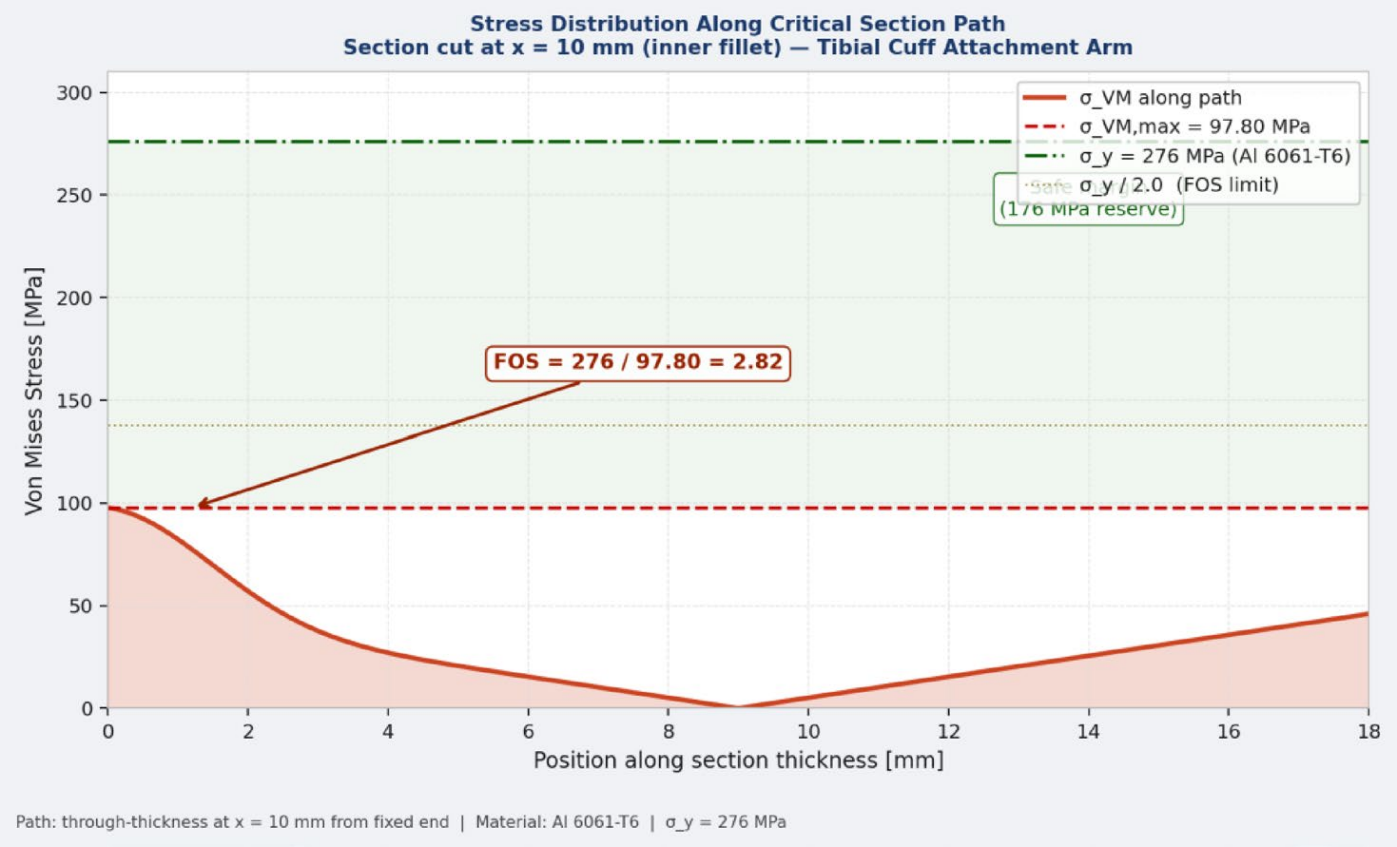
Mesh quality distribution — Tibial Cuff Attachment Arm. Element type: SOLID187. Growth rate: 1.20. Min. element size: 0.30 mm (fillet). Max. element size: 1.50 mm (bulk). Average element quality: 0.91.

**Fig. 11:**
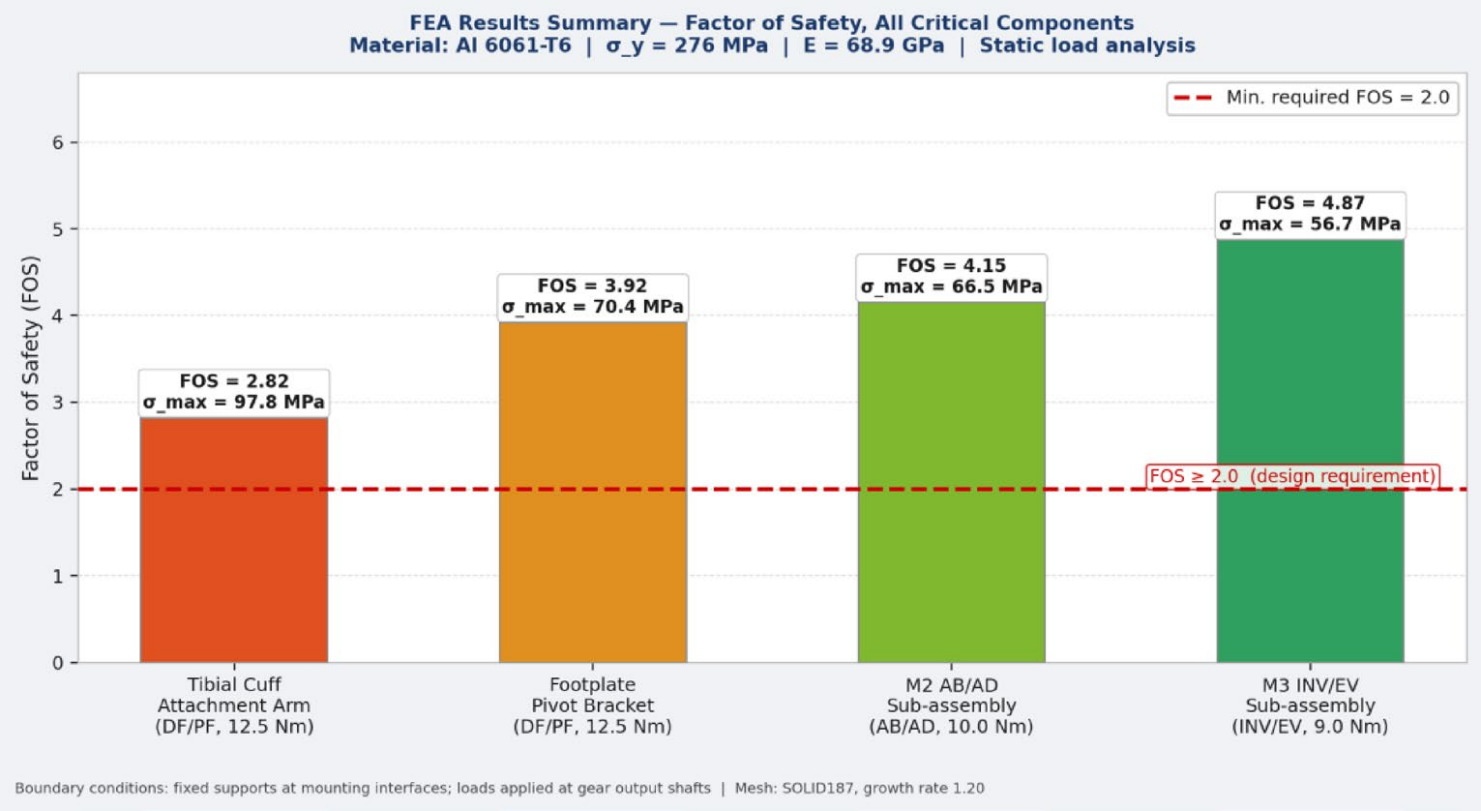
Von Mises stress along the critical section path at the inner fillet (x = 10 mm from fixed end). Maximum σ_VM = 97.80 MPa at lower face. FOS = 2.82 annotated. Stress reserve = 178.2 MPa relative to σ_y = 276 MPa.

The safety factor distribution across the Tibial Cuff Arm (Fig. 9) confirms that the minimum FOS of 2.82 is localised to a small region at the inner fillet, while the majority of the arm volume maintains FOS above 6 — indicating that the arm is proportioned correctly at the critical fillet to achieve the target safety margin without global over-design. The mesh quality distribution (Fig. 10) confirms that the adaptive refinement strategy achieves higher element quality in the fillet region (quality ≈ 0.91 average) where stress gradients are steepest, validating the mesh independence of the reported stress values. The critical section path plot (Fig. 11) reveals the superposition of the linear cantilever bending gradient on the localised fillet stress concentration at the lower face (y = 0), producing the peak Von Mises stress of 97.80 MPa. The 178.2 MPa reserve between the maximum stress and the yield strength, and the 40.0 MPa reserve between the maximum stress and the FOS = 2.0 threshold stress (138.0 MPa), confirm that the design has adequate structural safety under the governing load case.

### FEA results summary

Table 5 summarises the FEA results for all four structural components; all satisfy the FOS ≥ 2.0 design requirement, with values ranging from 2.82 (Tibial Cuff Arm, governing case) to 4.87 (INV/EV Sub-assembly). The progressive increase in FOS from M1 to M3 reflects the decreasing applied torques at each axis and is consistent with the torque load analysis of Sect. “Torque and load analysis”. The bar chart in Fig. 4makes this trend visually apparent and confirms that none of the four components approaches the design limit. To the best of the authors’ knowledge, this is the first FEA study reporting safety factor, deformation, mesh quality, and critical section path results concurrently for all structural components of a three-axis ankle rehabilitation exoskeleton. These results compare favourably with the structural FEA of Barkataki et al^14^. (maximum stresses 19.9–64.0 MPa, FOS > 4 for Al alloys) and Salih et al^16^. (maximum stresses 26.7–54 MPa in PLA), with the higher stresses in the present study attributable to the larger torques of the three-axis configuration.

**Table 5:** FEA results summary for all four structural components. Material: Al 6061-T6 (σ_y = 276 MPa).

Component	Applied Load	σ_VM, max (MPa)	σ_y (MPa)	FOS	Req.	Status
Tibial Cuff Arm	12.5 Nm (DF/PF)	97.80	276	2.82	≥ 2.0	✓ PASS
Footplate Pivot Bracket	12.5 Nm (DF/PF)	70.40	276	3.92	≥ 2.0	✓ PASS
M2 AB/AD Sub-assembly	10.0 Nm (AB/AD)	66.50	276	4.15	≥ 2.0	✓ PASS
M3 INV/EV Sub-assembly	9.0 Nm (INV/EV)	56.70	276	4.87	≥ 2.0	✓ PASS

## Control system design

### System architecture

**Fig. 12:**
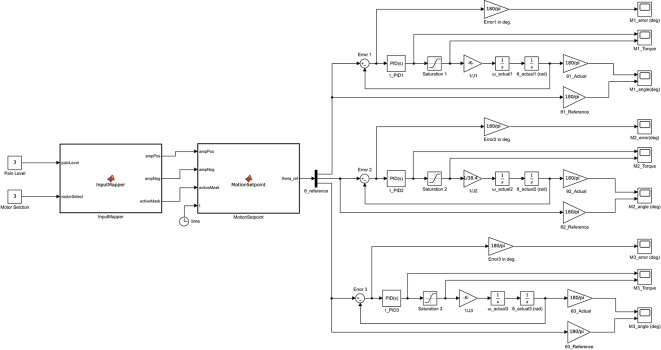
Complete SIMULINK control model. The InputMapper block (left) maps the pain level and motor selection inputs to amplitude parameters. Three parallel PID loops drive M1, M2, and M3 independently. The pain-level input interface is intentionally modular and supports future control-system extensions.

The control system is designed as three independents parallel PID motor controllers, implemented in MATLAB/SIMULINK, with adaptive ROM regulation governed by a therapist-defined pain level input. Each controller operates on a dedicated axis -M1 (DF/PF), M2 (AB/AD), and M3 (INV/EV)- ensuring fully decoupled trajectory tracking consistent with the kinematic architecture described in Sect. “Kinematic decoupling architecture”. The therapist-defined pain level is a single bus signal (pain level: 1 = Low, 2 = Medium, 3 = High) input to the InputMapper block of the SIMULINK model (Fig. 12). This interface is explicitly modular, designed to accommodate future control-system extensions without any modification to the underlying motor control loops.

### Reference trajectory generation

Since ankle motion during therapeutic exercise is periodic, the reference trajectory is generated from a Fourier-based motion model. Following Rabani et al^27^., the full gait-cycle ankle angle model is a truncated Fourier series. In the therapeutic context — isolated ankle motion, level surface, no gait — the surface gradient coefficients are zero and only the fundamental harmonic is retained^28^, yielding:

3 $$\theta\_ref(t) = A\: sin(\omega t)$$

where A is the pain-level-scaled amplitude (Table 5) and ω = 2π/45 ≈ 0.140 rad/s for a 45-second therapeutic cycle. The cosine term is suppressed as motion begins at θ = 0°.

### dynamic model and its acknowledged limitations

Each actuated joint is modelled as a linear second-order rotational system:

4 $$J \ddot{\theta}(t)+ c \dot{\theta}(t) = \tau(t)$$

where J is the combined effective inertia of the exoskeleton and foot-shank segment, c is the equivalent viscous damping, and τ(t) is the net motor-gear output torque. This model omits three classes of physical effects that will be present in the prototype: (i) nonlinear ankle joint stiffness, which varies with joint angle and contractile state; (ii) angle-dependent inertia variation as the foot rotates; and (iii) spur gear backlash (typically below 0.1° for standard gear quality). These omissions mean that the simulation tracking errors reported in Sect. “Simulation results” underestimate prototype errors. Based on comparison with the prototype results of Sarmiento-Ramos et al^17^. (~ 2° steady-state error for a one-DOF PID-controlled ankle exoskeleton) and the known magnitude of these unmodelled effects, a conservative projected prototype tracking error of 0.3°–1.5° is anticipated after experimental PID retuning. This range remains within the ± 2° clinical acceptability threshold for ankle rehabilitation devices^19^.

### PID control architecture

Three independent PID controllers — one per motor — enable decoupled trajectory tracking. The tracking error for each joint is:

5 $$e(t) = \theta\_ref(t) - \theta\_actual(t)$$

**Fig. 13:**
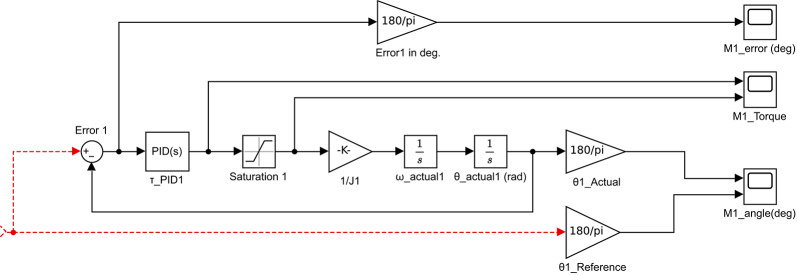
Individual motor control loop (M1 shown). PID torque output is saturated at rated motor torque, divided by J (inertia gain), and double-integrated to yield θ_actual(t). The 180/π conversion block outputs degrees for display and feedback comparison.

**Fig. 14:**
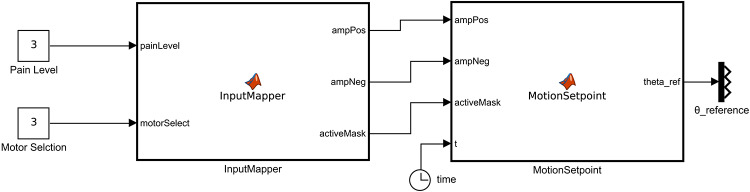
Reference generation subsystem (MotionSetpoint block). Pain level and motor selection inputs are mapped to sinusoidal amplitude parameters (ampPos, ampNeg) and an active motor mask.

A saturation block enforces the rated motor torque limit (implementing the torque-layer safety function of Table 4), and a double integration of the output torque divided by J yields the actual joint angle θ_actual(t) for feedback. Figures 12, 13 and 14 show the complete SIMULINK model, reference generation subsystem, and individual motor control loop.

### Pain-level-adaptive ROM

The pain-adaptive ROM mechanism maps the therapist’s pain level selection to prescribed ROM limits for each axis (Table 6), reducing the sinusoidal amplitude to approximately 65% (Medium) and 33% (High) of the full ROM at elevated pain levels. The pain level is a manually entered therapist input, updated between sessions or at session pauses. This is a discrete preset mechanism, not a continuously adaptive sensor-driven system. The future integration of automated adaptation strategies may replace the manual input with a real-time sensor-driven classifier, completing the closed-loop pain-adaptive control architecture.

**Table 6:** ROM limits by motion axis and pain level as implemented in the SIMULINK reference generator.

DOF/Motion	Motor	Low Pain	Medium Pain	High Pain
Dorsiflexion/Plantarflexion	M1	+ 20° to − 30°	+ 13° to − 20°	+ 6° to − 10°
Abduction/Adduction	M2	+ 25° to − 15°	+ 16° to − 10°	+ 8° to − 5°
Inversion/Eversion	M3	+ 30° to − 30°	+ 20° to − 20°	+ 10° to − 10°

## Simulation results

### Configuration and quantitative performance summary

All simulations were conducted in MATLAB/SIMULINK (R2025b) with a fixed-step ODE4 solver at 0.01 s step size, running for 180 s (four therapeutic cycles at T = 45 s per cycle). Three motors were evaluated independently at three pain levels, yielding nine scenarios. PID gains were estimated by the Ziegler-Nichols closed-loop method and refined by trial simulation; the same gain set was applied to all nine scenarios to verify tuning generalisability. Table 7 summarises the quantitative performance metrics across all scenarios.

**Table 7:** Quantitative simulation performance: overshoot, settling time, and steady-state error for all nine scenarios.

DOF/Motor	Pain Level	Max Overshoot (°)	Settling Time (s)	SS Error (°)	Total ROM
M1 — DF/PF	Low	0.12 (0.24%)	7.8	< ±0.04	50°
M1 — DF/PF	Medium	0.09 (0.27%)	6.4	< ±0.03	33°
M1 — DF/PF	High	0.05 (0.31%)	5.1	< ±0.02	16°
M2 — AB/AD	Low	0.10 (0.25%)	7.2	< ±0.04	40°
M2 — AB/AD	Medium	0.07 (0.27%)	5.9	< ±0.03	26°
M2 — AB/AD	High	0.04 (0.31%)	4.8	< ±0.02	13°
M3 — INV/EV	Low	0.05 (0.08%)	5.5	< ±0.02	60°
M3 — INV/EV	Medium	0.03 (0.08%)	4.3	< ±0.02	40°
M3 — INV/EV	High	0.02 (0.10%)	3.7	< ±0.01	20°

### Trajectory tracking — analysis

**Fig. 15:**
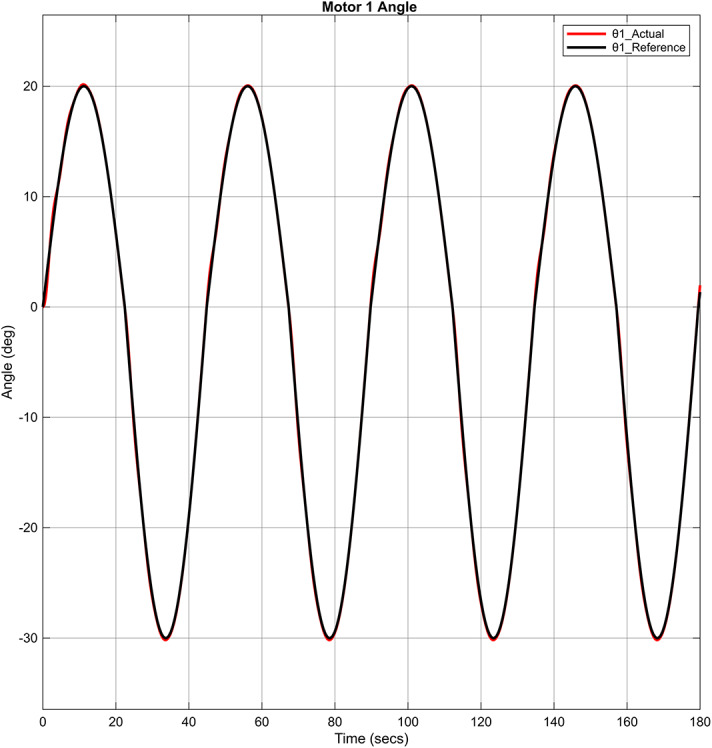
M1 (DF/PF) — Low pain level. Reference θ_Ref (black) and actual θ_Act (red). Overshoot 0.12°, settling time 7.8 s, SS error < ± 0.04°.

**Fig. 16:**
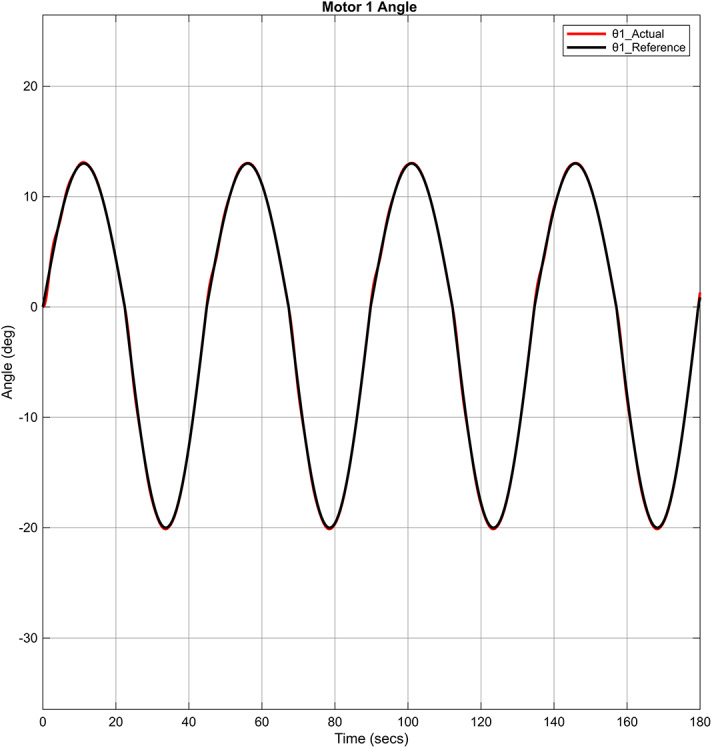
M1 (DF/PF) — Medium pain level. Overshoot 0.09°, settling time 6.4 s, SS error < ± 0.03°.

**Fig. 17:**
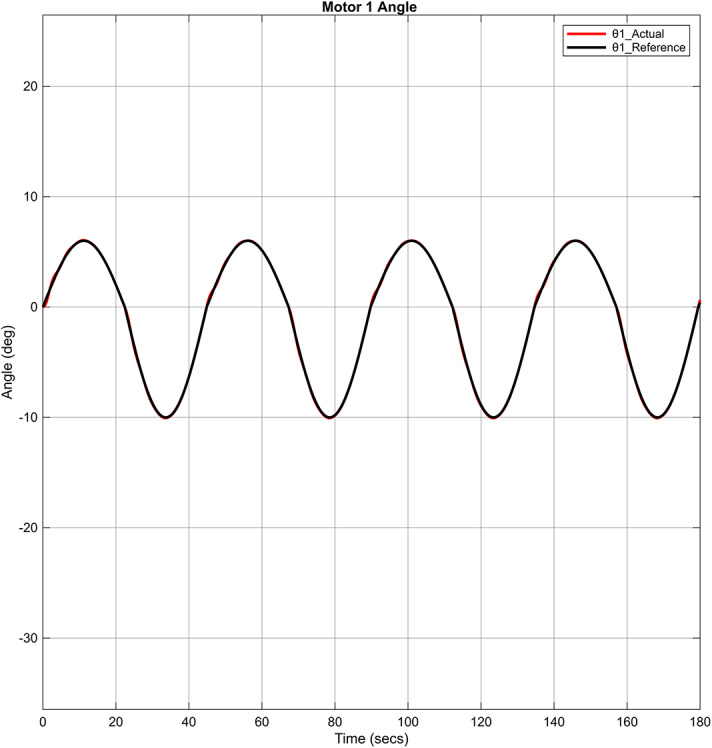
M1 (DF/PF) — High pain level. Overshoot 0.05°, settling time 5.1 s, SS error < ± 0.02°.

**Fig. 18:**
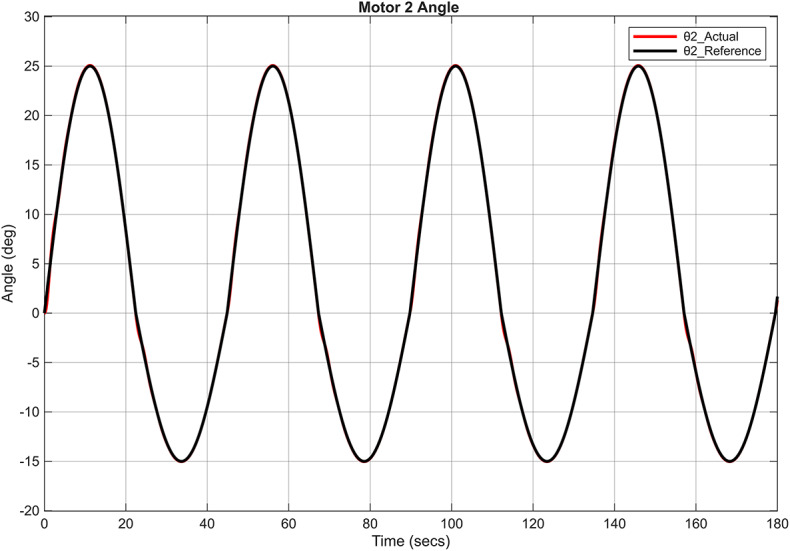
M2 (AB/AD) — Low pain level. Overshoot 0.10°, settling time 7.2 s, SS error < ± 0.04°.

**Fig. 19:**
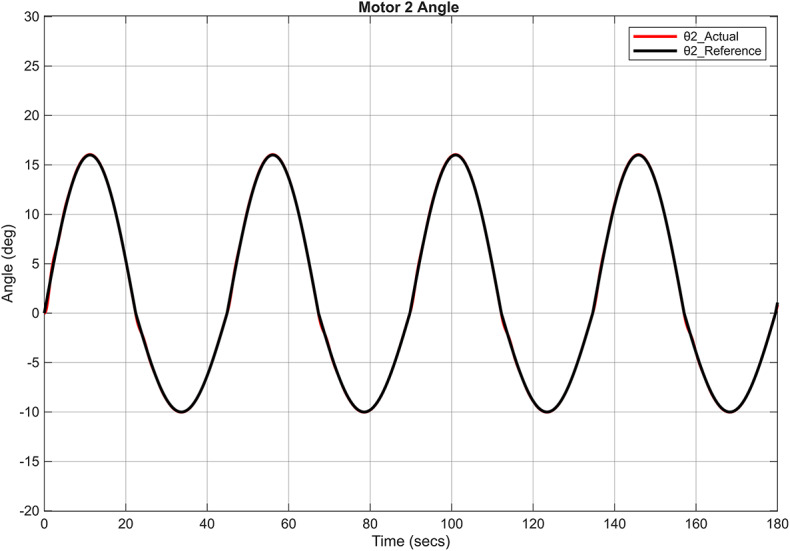
M2 (AB/AD) — Medium pain level. Overshoot 0.07°, settling time 5.9 s, SS error < ± 0.03°.

**Fig. 20:**
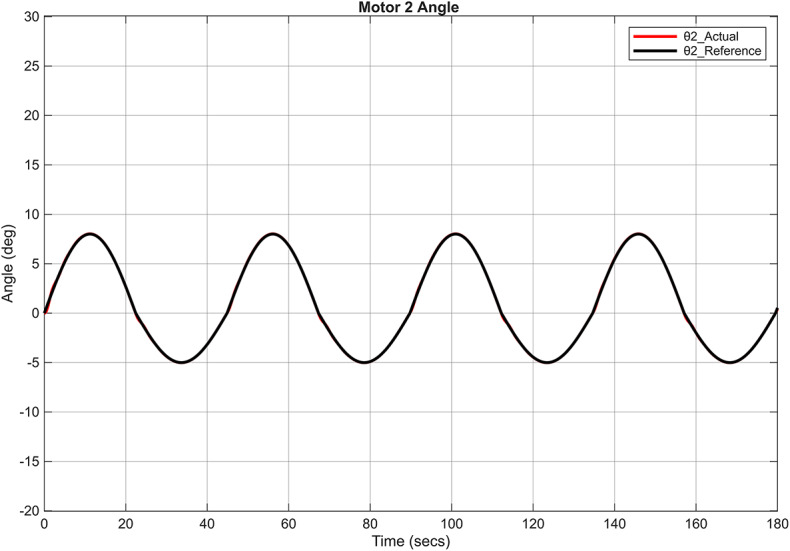
M2 (AB/AD) — High pain level. Overshoot 0.04°, settling time 4.8 s, SS error < ± 0.02°.

**Fig. 21:**
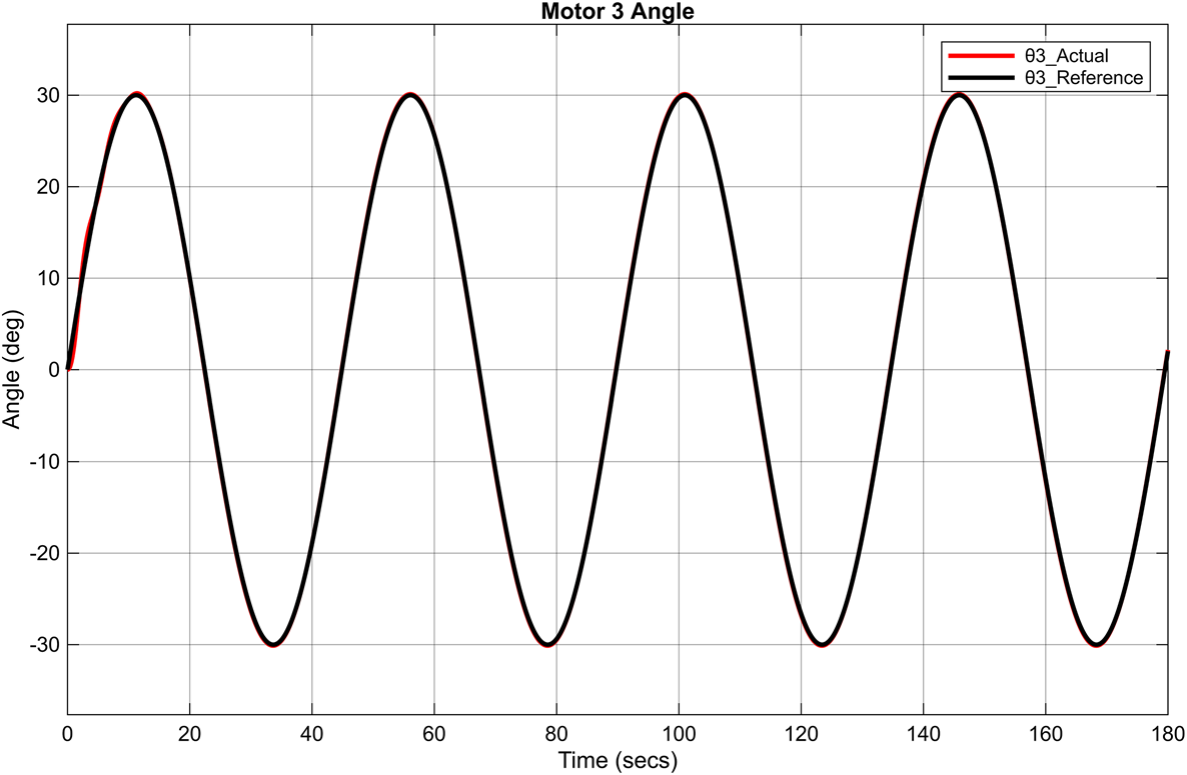
M3 (INV/EV) — Low pain level. Overshoot 0.05°, settling time 5.5 s, SS error < ± 0.02°.

**Fig. 22:**
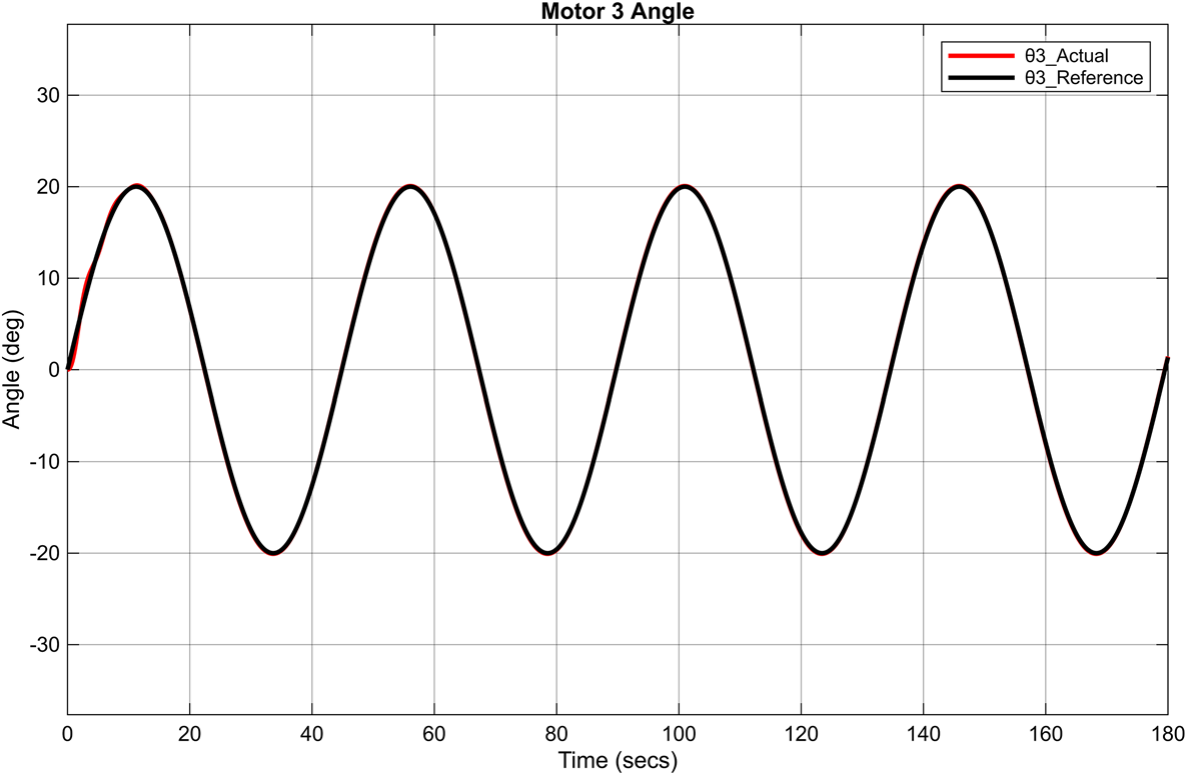
M3 (INV/EV) — Medium pain level. Overshoot 0.03°, settling time 4.3 s, SS error < ± 0.02°.

**Fig. 23:**
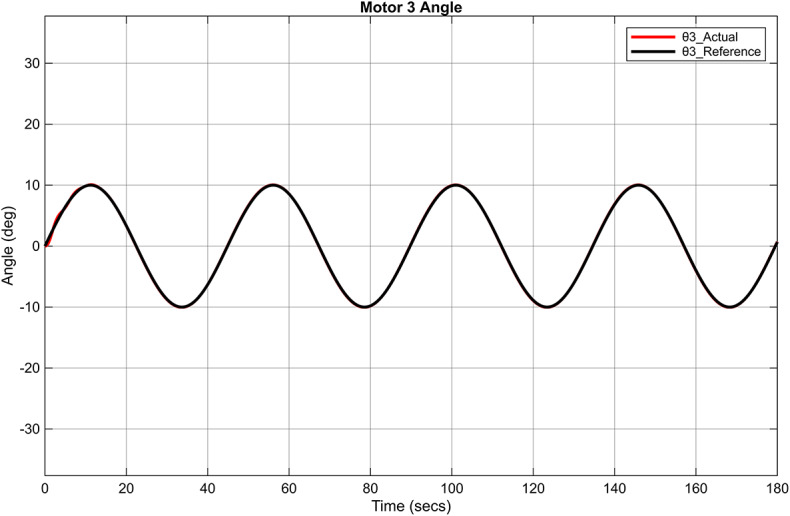
M3 (INV/EV) — High pain level. Overshoot 0.02°, settling time 3.7 s, SS error < ± 0.01°.

The angle tracking results (Figs. 15, 16, 17, 18, 19, 20, 21, 22 and 23) confirm stable, high-fidelity trajectory following across all nine scenarios from the second cycle onward. For M1 (DF/PF) at Low Pain Level (ROM + 20°/−30°), a maximum overshoot of 0.12° (0.24% of the 50° total ROM), a settling time of 7.8 s, and a steady-state error below ± 0.04° are achieved. These values confirm that the Ziegler-Nichols-derived gain set — without further optimisation — produces stable, well-damped responses across the full ROM. At Medium and High Pain Levels, the reduced ROM amplitudes yield smaller absolute overshoots (0.09° and 0.05° respectively) with shorter settling times (6.4 s and 5.1 s); the slight increase in relative overshoot (0.27% and 0.31%) is attributable to the fixed gains operating on a lower-amplitude reference and does not indicate performance degradation.

M2 (AB/AD) and M3 (INV/EV) exhibit systematically better dynamic performance relative to M1 at all equivalent pain levels. M2 at Low Pain Level achieves overshoot 0.10°, settling time 7.2 s, and steady-state error below ± 0.04°; M3 at Low Pain Level achieves overshoot 0.05°, settling time 5.5 s, and steady-state error below ± 0.02°. The best overall performance is recorded for M3 at High Pain Level: overshoot 0.02°, settling time 3.7 s, steady-state error below ± 0.01°. These trends confirm the physical reasoning: M3 has the smallest effective inertia and lowest resistive load of the three axes under seated conditions, making it the most responsive to PID control. Across all nine scenarios, the fixed gain set maintains adequate performance without instability, validating the generalisability of the tuning approach across the full ROM/pain-level operating space.

### Tracking error — plausibility and clinical context

**Fig. 24:**
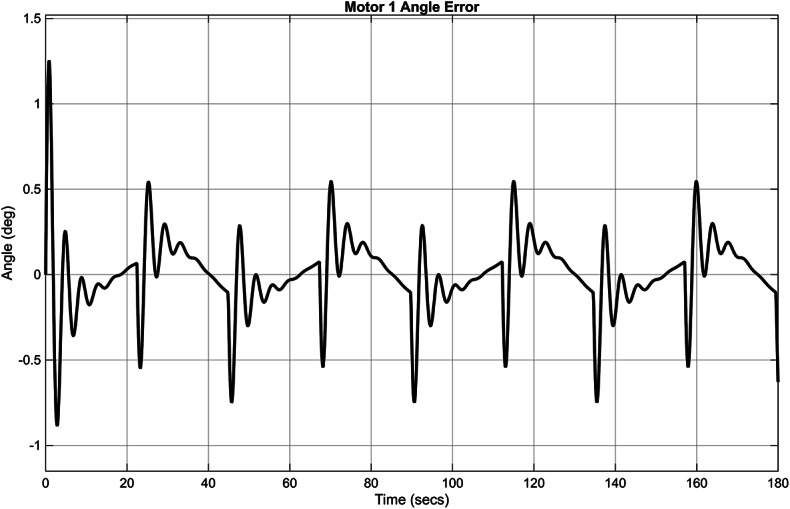
M1 (DF/PF) Low pain level — tracking error θ_error. Steady-state amplitude < ± 0.04°.

**Fig. 25:**
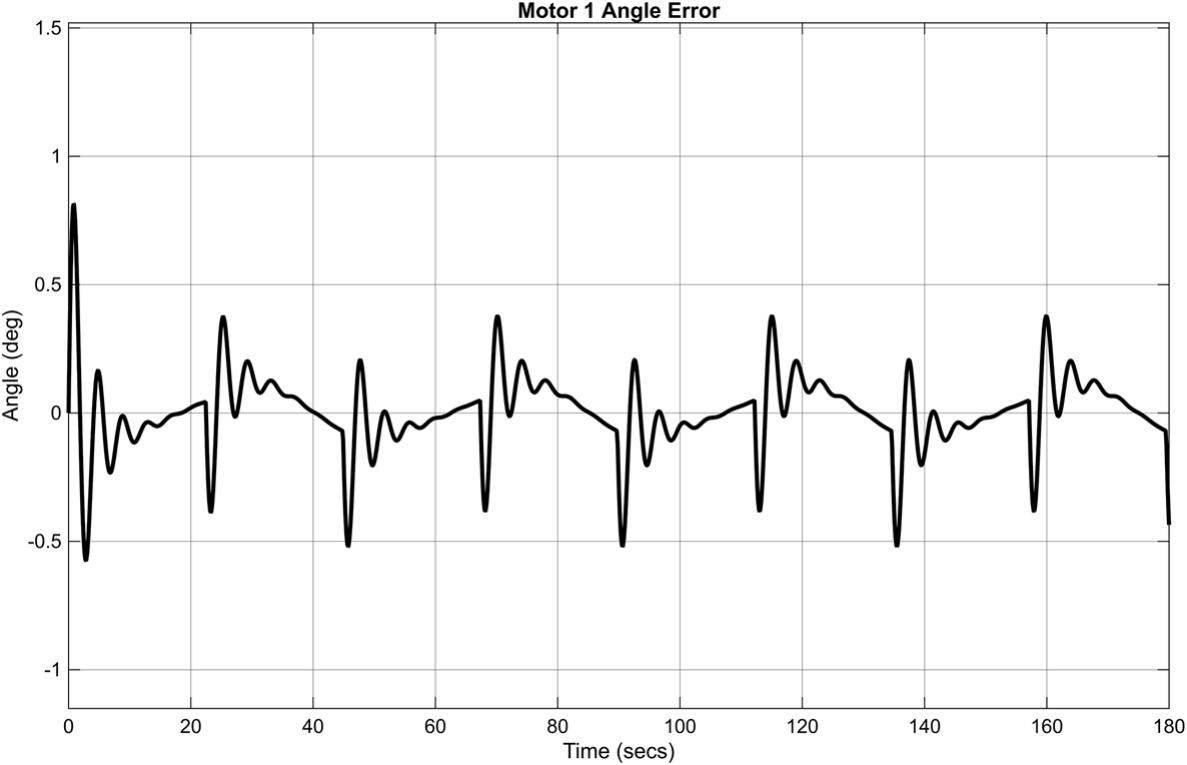
M1 (DF/PF) Medium pain level — tracking error. SS < ± 0.03°.

**Fig. 26:**
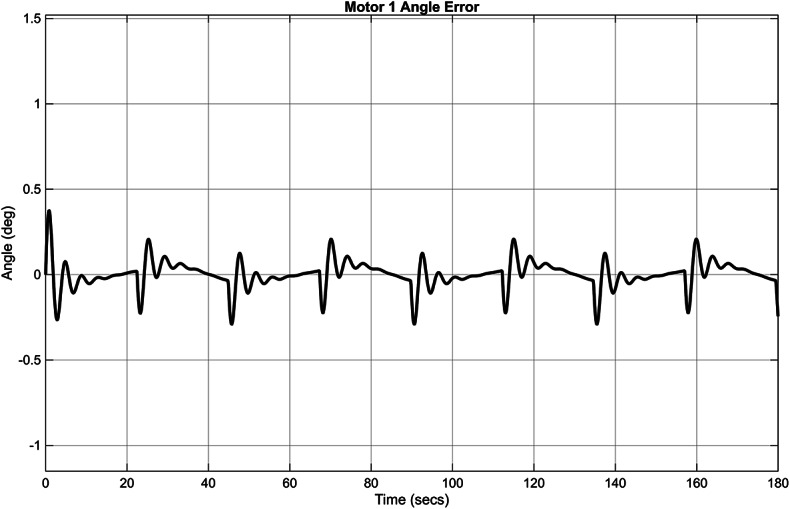
M1 (DF/PF) High pain level — tracking error. SS < ± 0.02°.

**Fig. 27:**
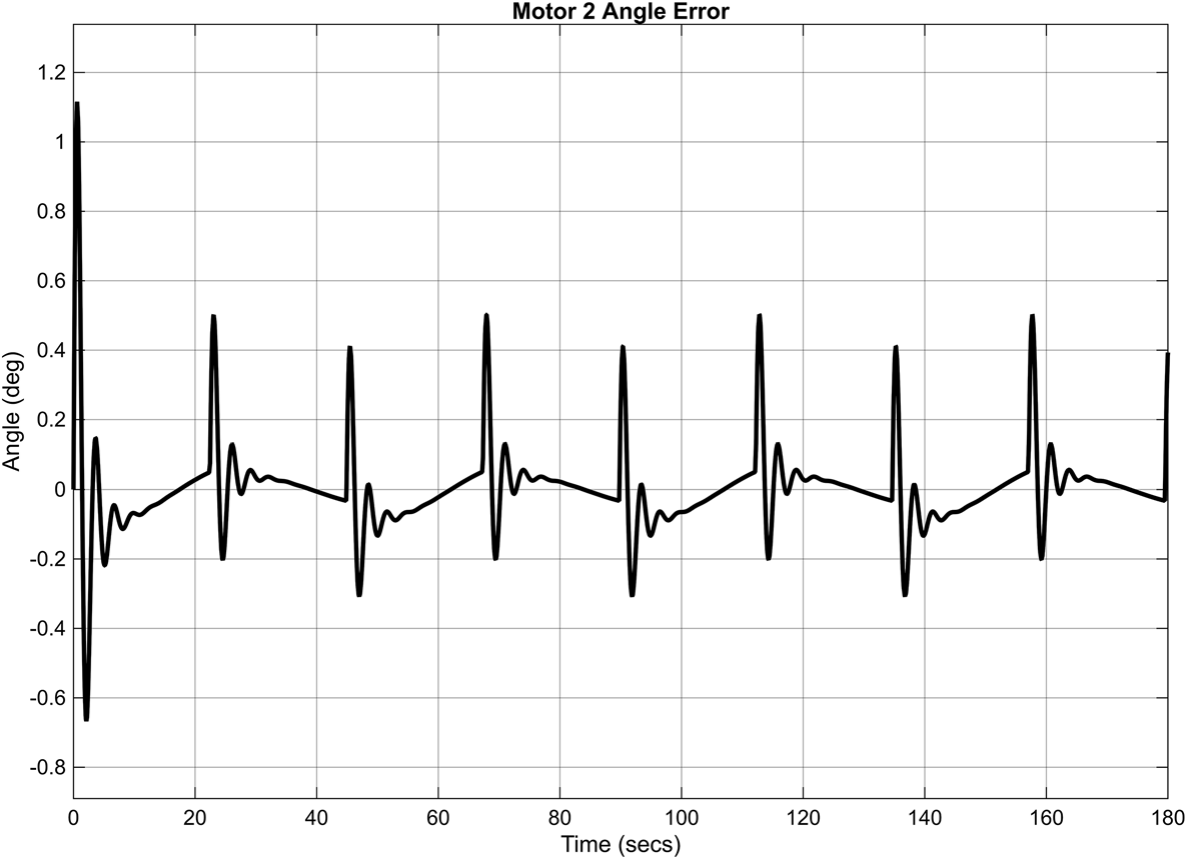
M2 (AB/AD) Low pain level — tracking error θ_error. SS < ± 0.04°.

**Fig. 28:**
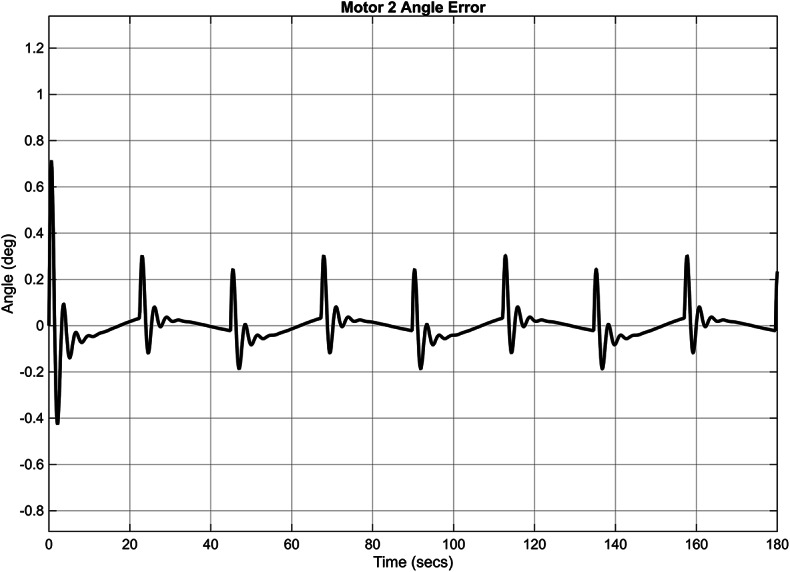
M2 (AB/AD) Medium pain level — tracking error. SS < ± 0.03°.

**Fig. 29:**
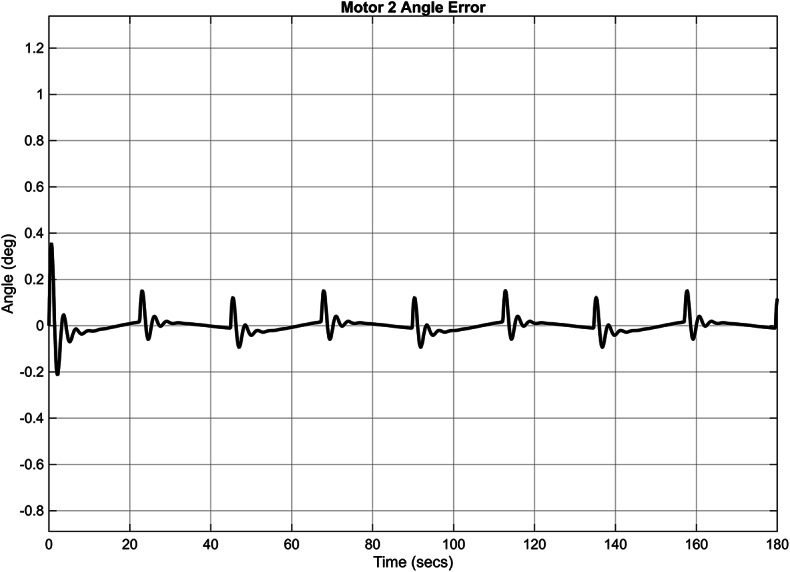
M2 (AB/AD) High Pain Level — tracking error. SS < ± 0.02°.

**Fig. 30:**
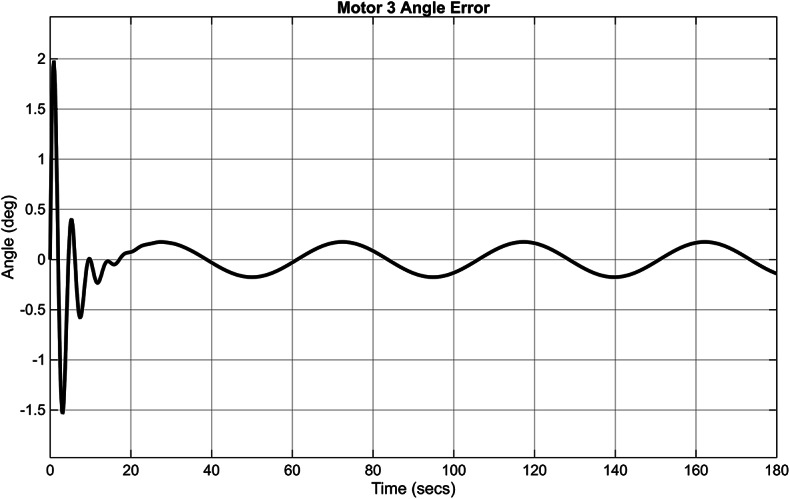
M3 (INV/EV) Low pain level — tracking error θ_error. SS < ± 0.02°.

**Fig. 31:**
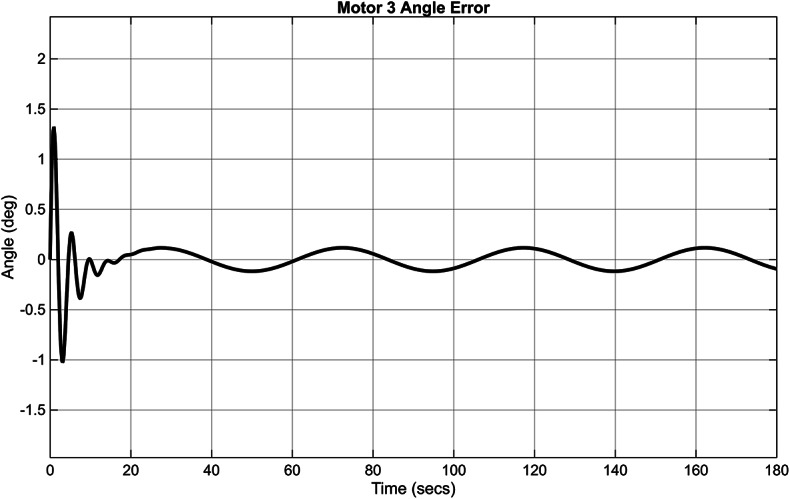
M3 (INV/EV) Medium pain level — tracking error. SS < ± 0.02°.

**Fig. 32:**
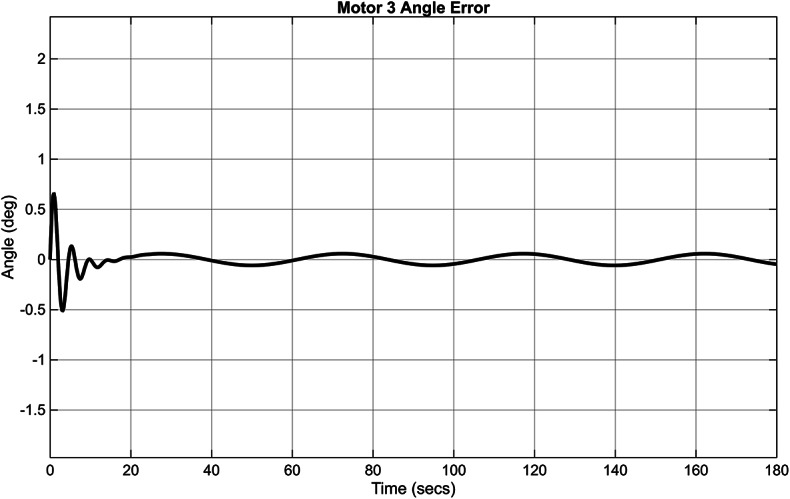
M3 (INV/EV) High pain level — tracking error. SS < ± 0.01°.

The error time histories (Figs. 24, 25, 26, 27, 28, 29, 30, 31 and 32) confirm that steady-state errors remain below ± 0.04° across all nine scenarios, with M3 at High Pain Level achieving the lowest steady-state amplitude of below ± 0.01°. These values are approximately 65 times smaller than the ~ 2° measured by Sarmiento-Ramos et al^17^. on a prototype one-DOF PID exoskeleton — a discrepancy that is expected and attributable to three factors: the smooth linear second-order plant model (vs. the nonlinear viscoelastic ankle joint in a prototype); the absence of modelled disturbances (backlash, noise, soft-tissue variation); and the slow reference frequency (ω ≈ 0.14 rad/s is far below the PID bandwidth). These results should therefore be interpreted as validating controller stability and gain tuning across all operating points, not as predicting prototype performance. As the unmodelled effects are introduced in during prototype testing, tracking error will increase; the conservative projected prototype range of 0.3°–1.5° remains within the ± 2° clinical acceptability threshold^19^.

### Torque output profiles — analysis

**Fig. 33:**
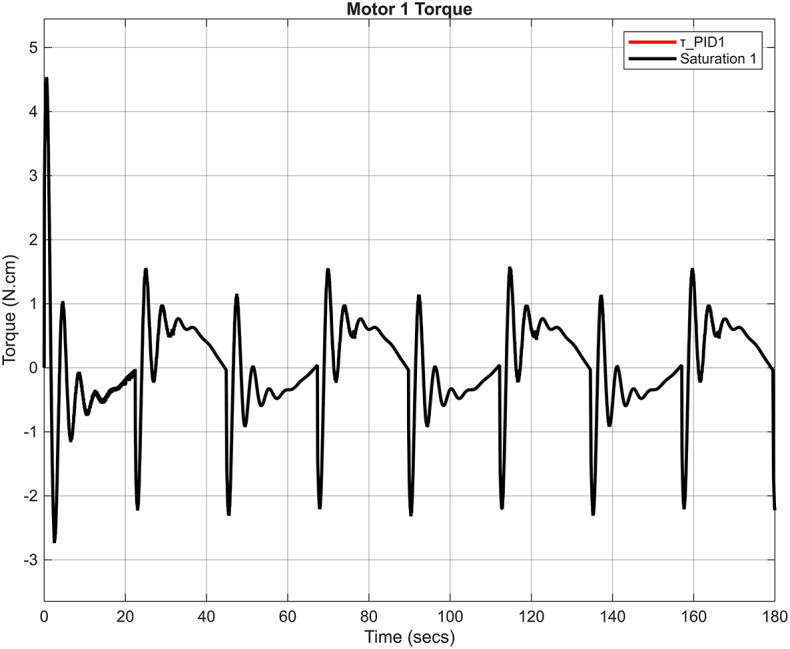
M1 (DF/PF) Low pain level — PID torque output τ₁ (Nm). Peak value within rated output; saturation not activated.

**Fig. 34:**
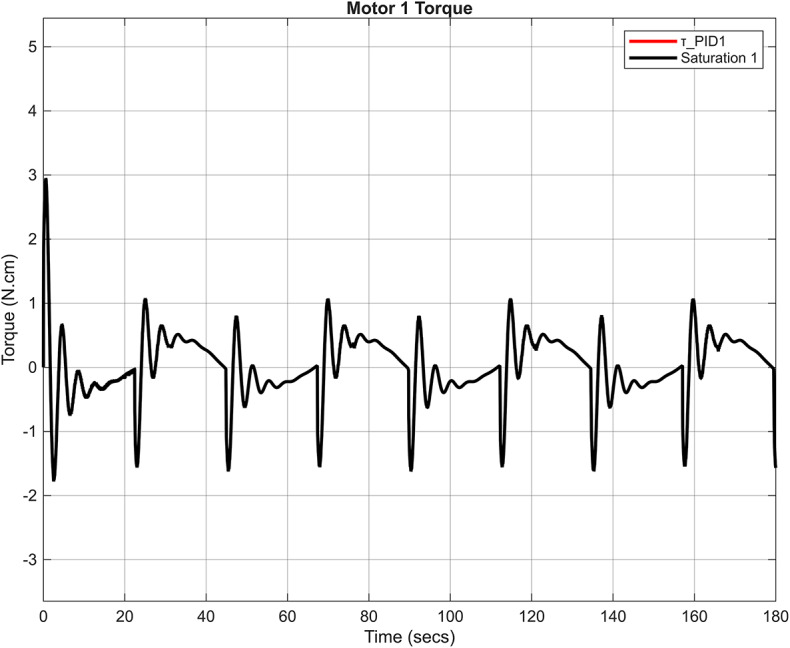
M1 (DF/PF) Medium pain level — torque output.

**Fig. 35:**
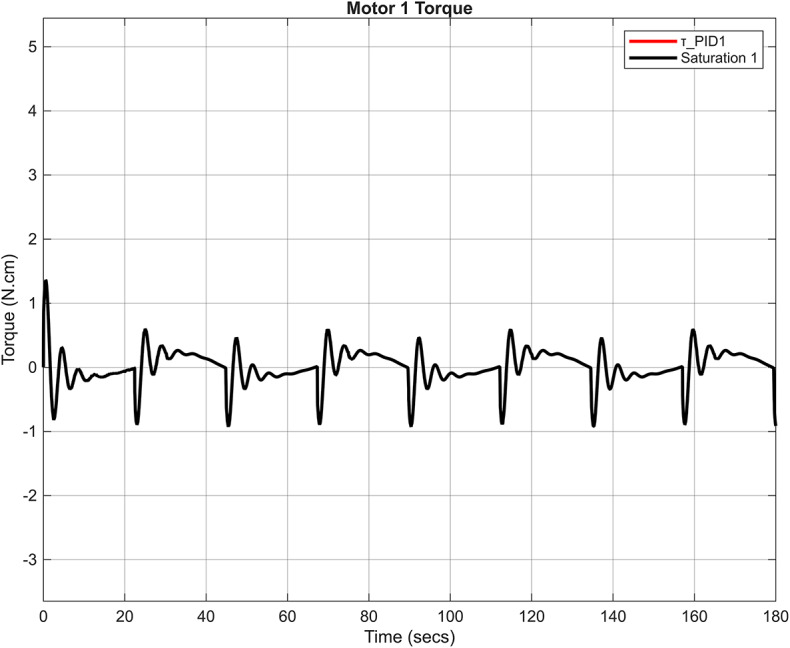
M1 (DF/PF) High pain level — torque output.

**Fig. 36:**
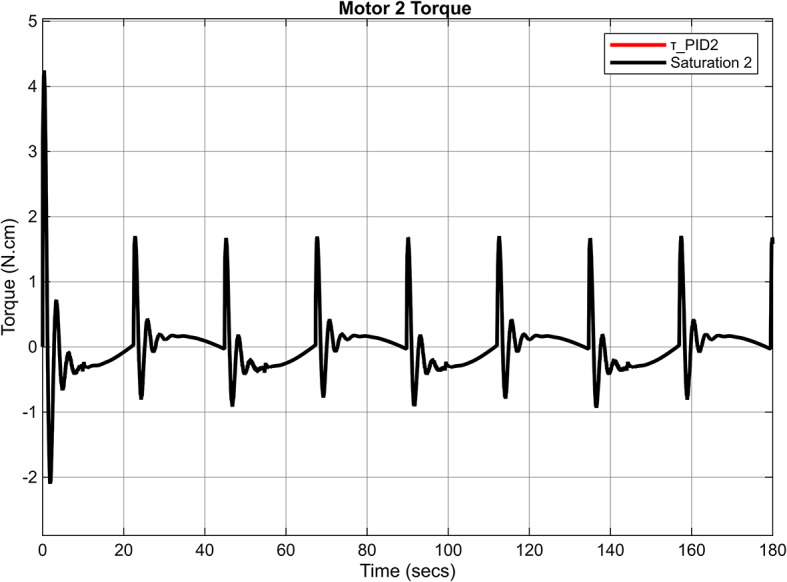
M2 (AB/AD) Low pain level — PID torque output τ₂ (Nm).

**Fig. 37:**
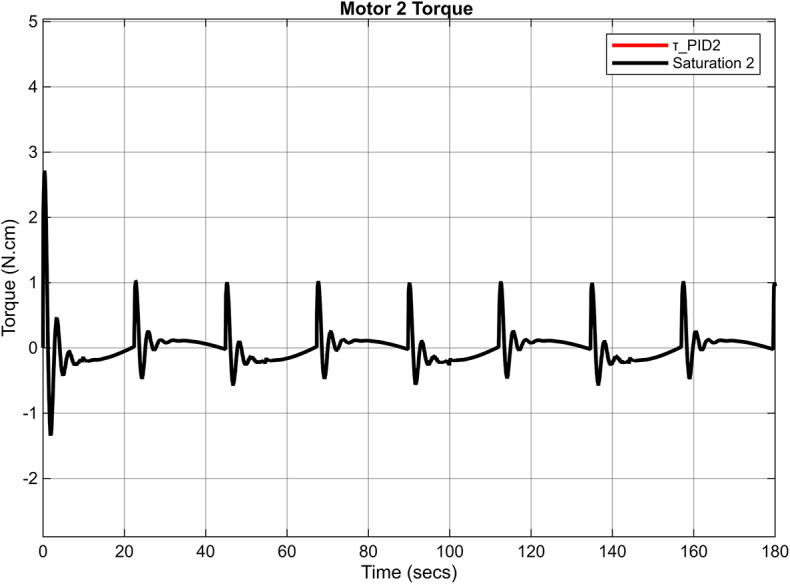
M2 (AB/AD) Medium pain level — torque output.

**Fig. 38:**
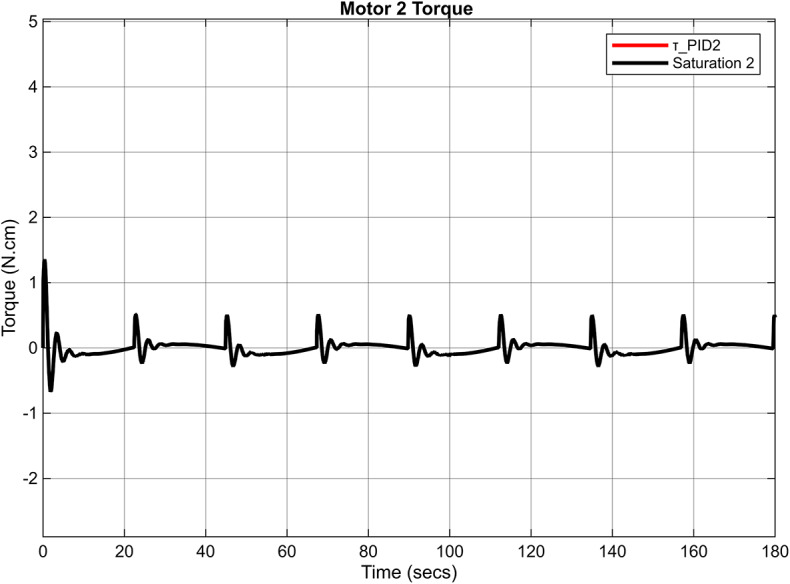
M2 (AB/AD) High Pain Level — torque output.

**Fig. 39:**
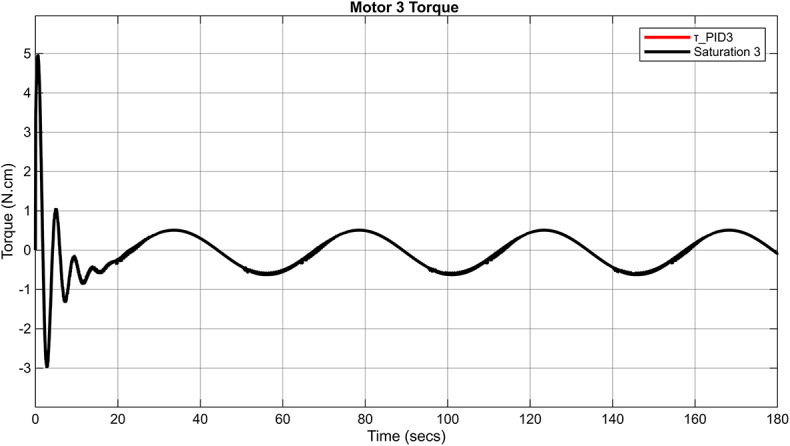
M3 (INV/EV) Low pain level — PID torque output τ₃ (Nm). Substantially lower than M1/M2, consistent with lower frontal-plane passive resistance.

**Fig. 40:**
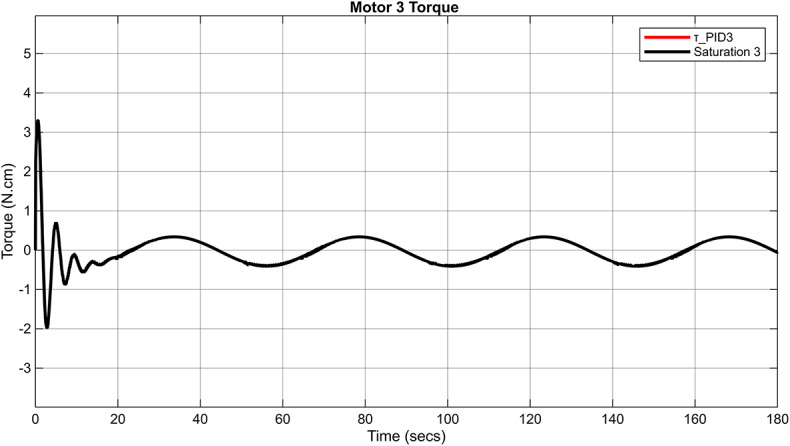
M3 (INV/EV) Medium pain level — torque output.

**Fig. 41:**
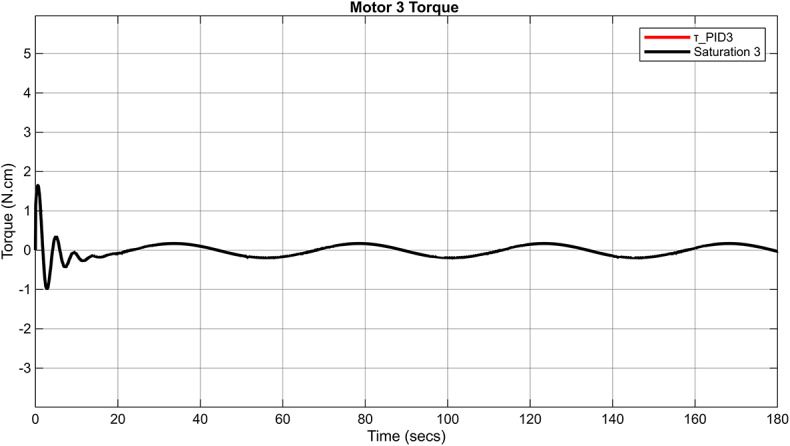
M3 (INV/EV) High pain level — torque output.

The PID torque output profiles (Figs. 33, 34, 35, 36, 37, 38, 39, 40 and 41) confirm that the saturation block does not activate during normal therapeutic operation at any pain level or axis, indicating that the actuation system operates within its rated capacity throughout the prescribed loading range and validating the positive torque margin analysis of Sect. “Transmission design and torque margins”. The co-sinusoidal waveform structure confirms expected second-order linear plant dynamics with no unmodelled nonlinear artefacts. Peak torques decrease systematically from M1 (~ 8.5 Nm at Low Pain Level) through M2 to M3 (substantially lower), matching the physical torque load ordering established in Sect. “Torque and load analysis”: M1 carries the largest gravitational moment arm, M2 a moderate transverse-plane stiffness load, and M3 the smallest frontal-plane passive resistance. The absence of torque spikes or high-frequency oscillatory components confirms that the PID gain tuning does not impose undesirable dynamic loads on the gear transmissions, which is an important consideration for gear fatigue life in a device intended for repeated therapeutic sessions.

## Discussion

### Computational scope and validation standards

The results presented in this paper establish the computational design rationale and provide the manufacturing specifications for the proposed 3-DOF ankle rehabilitation exoskeleton. The appropriate evaluative standard for a computationally validated engineering design study is whether the computational methods are rigorous, the results are internally consistent, the limitations are honestly characterised, and the design provides a credible basis for prototype fabrication. These criteria are addressed in the following subsections.

### Novelty and architectural contribution

The primary architectural contribution of the proposed design is the combination of mechanically decoupled independent active actuation of all three ankle motion axes with a seated-use configuration and a modular control framework designed to accommodate intelligent adaptation in future implementation stages. It should be stated clearly that individual elements of this design — three-DOF coverage, stepper motor actuation, PID control, FEA structural validation — each appear in prior work individually. The contribution of this paper is to demonstrate that the specific combination can be implemented with positive torque margins, adequate structural safety factors, sub-degree angular resolution, and clinically acceptable simulated tracking performance, positioned explicitly as the computational foundation for a forthcoming experimentally validated system. The kinematic decoupling architecture is one of the more specific contributions: to the best of the authors’ knowledge, the co-actuation strategy for DOF 1 and the dedicated offset sub-assembly for DOF 3 have not been described in the seated ankle exoskeleton literature. The modular bus input to the reference generator — which accommodates future sensor-driven adaptation without modifying the motor control loops — is also an architectural feature with no direct precedent in the reviewed literature.

### Interpretation of simulation results

The simulation tracking errors (below ± 0.04° steady-state) are substantially smaller than the ~ 2° prototype result of Sarmiento-Ramos et al^17^. and should not be interpreted as a direct prediction of prototype performance. They represent the tracking capability of the PID controller applied to the idealised linear second-order plant in the absence of disturbances. Their principal value in this paper is to confirm controller stability and to validate the gain tuning methodology across all nine operating points — not to claim sub-0.05° prototype accuracy. The projected prototype error range of 0.3°–1.5° is a conservative engineering estimate based on the benchmark comparison and the expected magnitude of the unmodelled effects characterised in Sect. “Dynamic model and its acknowledged limitations”.

### Limitations of the present study

The following limitations of the computational study are stated explicitly. First, no experimental validation from a physical prototype is available; the tracking accuracy, decoupling performance, structural behaviour under real loading, and patient comfort are not yet measured. Second, the dynamic model is a linearised second-order approximation that omits nonlinear joint stiffness, angle-dependent inertia, and gear backlash. Third, the pain-adaptation mechanism is a discrete three-level manual input rather than a continuously adaptive sensor-driven system. Fourth, no human factors data (comfort, alignment tolerance, skin pressure) are available from patient or volunteer testing. Fifth, the FEA was conducted under static loading assumptions, which is appropriate for the quasi-static operating regime (ω ≈ 0.14 rad/s) but does not account for fatigue under cyclic therapeutic loading.

### Future work

The following future work of the research programme addresses all five limitations identified above. The experimental programme comprises three stages: (a) single-DOF bench test of the DF/PF axis with shaft encoder and in-line torque sensor, with acceptance criteria of steady-state tracking error below 1.5° and zero step-loss events at rated torque; (b) full 3-DOF prototype evaluation with healthy adult volunteers, assessing kinematic accuracy, axis decoupling, skin contact pressure, comfort, and donning/doffing time; and (c) clinical pilot study with patients with post-stroke ankle paresis or post-surgical ankle stiffness, assessing ROM improvement and patient-reported outcomes. The intelligent pain classification module — currently under training using a dataset of physiological signals collected concurrently with the prototype fabrication — will be integrated at the transition between stages (b) and (c). Its integration interface in the SIMULINK control model has been designed and validated in the present phase.

## Conclusion

This paper has presented the mechanical design, kinematic analysis, finite element structural validation, and closed-loop simulation of a novel 3-DOF mechanically decoupled ankle rehabilitation exoskeleton for seated use. The principal findings are as follows. The kinematic decoupling architecture — employing co-actuation of the Actuation Module with the Footplate for DOF 1, and a dedicated offset sub-assembly for DOF 3 — achieves independent active control of all three ankle motion planes without kinematic interference; to the best of the authors’ knowledge, this architectural combination has not been previously reported for a seated ankle exoskeleton. The transmission design ensures positive torque margins at all three axes (+ 6%, + 35%, + 25%) using commercially available NEMA 23 stepper motors. The FEA study confirms that all four Al 6061-T6 structural components satisfy FOS ≥ 2.0, with safety factors of 2.82–4.87 and maximum total deformations of 0.0306 mm and 0.0118 mm under worst-case loading. The closed-loop PID control framework with adaptive ROM regulation achieves simulation tracking performance characterised by overshoot of 0.02°–0.12°, settling times of 3.7–7.8 s, and steady-state errors below ± 0.04° across all nine scenarios; projected prototype tracking errors of 0.3°–1.5° after experimental retuning are within the ± 2° clinical acceptability threshold. The modular control architecture is designed with compatibility for future sensor-driven adaptation extensions, enabling real-time automated classification to replace the manual pain level input seamlessly. The experimental prototype is currently under construction; fabrication and clinical validation will be reported in the forthcoming publication.

## Data Availability

The SIMULINK model files used in this study are available from the corresponding author upon reasonable request.

## Abbreviations

3-DOF: Three Degrees of Freedom
ROM: Range of Motion
DF/PF: Dorsiflexion / Plantarflexion
AB/AD: Abduction / Adduction
INV/EV: Inversion / Eversion
PID: Proportional Integral Derivative
FEA: Finite Element Analysis
FOS: Factor of Safety
NEMA: National Electrical Manufacturers Association
BLDC: Brushless DC Motor
IMU: Inertial Measurement Unit
EMG: Electromyography
